# Li‐Well ZnO Memtransistors: High Reliability for Neuromorphic Applications

**DOI:** 10.1002/adma.202506128

**Published:** 2025-09-10

**Authors:** Ki‐Hoon Son, Hyun‐Sik Kim, Dae‐Hee Han, Hyung‐Kyu Lim, Hong‐Sub Lee

**Affiliations:** ^1^ Department of Materials Science & Engineering Kyung Hee University Yongin 17104 Republic of Korea; ^2^ Department of Chemical Engineering Interdisciplinary Program in Advanced Functional Materials and Devices Development Kangwon National University 1 Kangwondaehak‐gil Chuncheon Gangwon 24341 Republic of Korea

**Keywords:** crossbar array, high reliability, lithium well, memtransistor, oxide semiconductor

## Abstract

Memtransistors are active analog memory devices utilizing ionic memristive materials as channel layers. Since their introduction, the term “memtransistor” has widely been adopted for transistors exhibiting nonvolatile memory characteristics. Currently, memtransistor devices possessing both transistor on/off functionality and nonvolatile memory characteristics include ferroelectric field‐effect transistors (FeFETs) and charge‐trap flash (floating gate), yet ionic memtransistors have not matched their performance. Here a facile and extendable lithium (Li)‐well oxide memtransistor (LWOM) is reported as a promising candidate. Forming a Li well, analogous to an n^+^ well beneath electrodes in n‐metal‐oxide‐semiconductor field‐effect transistor (MOSFET) processes, induces Li⁺‐ion migration via write *V*_DS_, achieving analog memory characteristics through Schottky barrier modulation. LWOM enables low‐voltage weight updates and precise gate‐controlled weight update characteristics. Analysis via 3D secondary ion mass spectrometry (SIMS) confirms Li‐ion redistribution and the resistance‐switching mechanism. A 21 × 21 crossbar array demonstrates 99.31% operational yield and successful weight updates to target conductance values. Fabricated using mature oxide semiconductor technology with a 230 °C thermal budget and a simple process, LWOM stands as a strong contender for next‐generation nonvolatile memory and artificial neural network (ANN) acceleration hardware.

## Graphical abstract

A novel lithium (Li)‐well ZnO memtransistor integrates transistor switching and nonvolatile memory, akin to ferroelectric field‐effect transistors (FeFETs) and floating gates, while enabling low‐power operation via Li⁺ ion migration. Li⁺ ion migration and switching mechanisms were confirmed by 3D secondary ion mass spectrometry (SIMS). A 21 × 21 crossbar array achieves 99.31% yield, and this scalable design supports neuromorphic computing and artificial neural network accelerators.

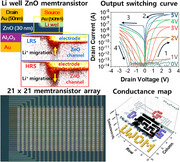

## 1 Introduction

Since the 2008 report “The Missing Memristor Found,”^[^ ^1^ ^]^ the term “memristor” has been broadly used to describe two‐terminal analog memory devices,^[^ ^2^, ^3^, ^4^, ^5^ ^]^ such as ionic, phase‐change, ferroelectric, and spintronic memristors. Similarly, the term “memtransistor” has been widely adopted to refer to transistors with analog memory functionality since its introduction in 2018,^[^ ^6^ ^]^ including floating‐gate MOSFETs, charge‐trap flash (CTF), ferroelectric field‐effect transistors (FeFETs), and electrolyte‐gated devices.^[^ ^7^, ^8^, ^9^, ^10^ ^]^ Currently, floating‐gate (or CTF) and FeFET devices utilize the gate as the primary programming terminal to adjust threshold voltage while retaining transistor on/off functionality.^[^ ^11^, ^12^, ^13^ ^]^ In contrast, ionic memtransistors employ the drain as the main programming terminal for weight updates, while the gate voltage can modulate ion migration.^[^ ^14^ ^]^ This makes them a potential solution for overcoming challenges of two‐terminal memristors in crossbar array architectures (CAAs) for energy‐efficient vector‐matrix multiplication (VMM) in next‐generation artificial neural network (ANN) accelerators (Figure S1, Supporting Information). These challenges include stochastic ion migration, nonlinear/asymmetric weight updates, stuck states due to defect interactions, sneak currents, and difficulties in analog input operations due to nonlinear *I*–*V* characteristics.^[^ ^15^, ^16^, ^17^, ^18^ ^]^ However, the high migration barrier of ions (typically 0.8–1.0 eV) necessitates significantly high writing voltages to drain for weight update,^[^ ^19^ ^]^ limiting their competitiveness with FeFETs and CTF devices. The longer channel lengths exacerbate this compared to the 10–20 nm vertical structures of two‐terminal ionic memristors.

In this article, we report a highly reliable memtransistor utilizing an oxide semiconductor with a lithium (Li) well, demonstrating its performance in a 21 × 21 array. The Li‐well oxide memtransistor (LWOM) is a thin‐film transistor (TFT) with analog nonvolatile memory characteristics, allowing gate‐controlled on/off switching while supporting weight updates with low *V*_DS_ (1–3 V). During write operations, *V*_GS_ controls the weight update rate and saturation point, while read operations enable control over *I*_read_, conductance ratio, and current difference. Additionally, the direction of weight updates (potentiation or depression) can be switched based on the Li well's position relative to the drain or source, exhibiting complementary memristive characteristics.

**Figure 1:**
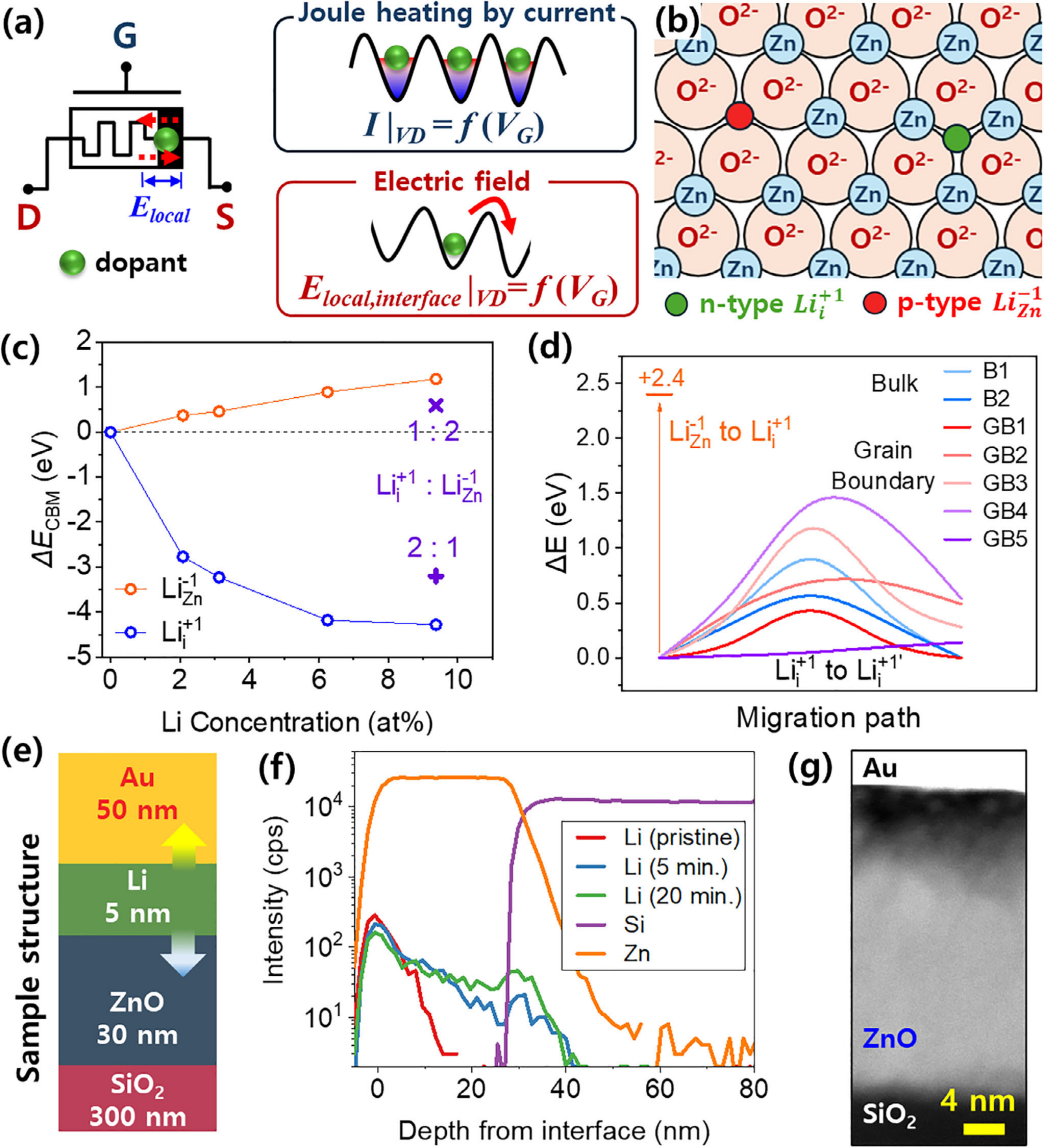
Role of doped Li in ZnO and Li‐well fabrication. a) Electrical symbol of the ionic memtransistor device (left) and schematic illustrating the two primary driving forces of ion migration responsible for memristive characteristics. b) Schematic depicting an n‐type defect ${\mathrm{Li}}_{i}^{+1}$ (green ball) occupying an interstitial site and a p‐type defect ${\mathrm{Li}}_{\mathrm{Zn}}^{-1}$ (red ball) occupying a Zn site within the ZnO crystal. c) Variation of conduction band minimum (CBM) energy relative to the Fermi level as a function of ${\mathrm{Li}}_{i}^{+1}$ and ${\mathrm{Li}}_{\mathrm{Zn}}^{-1}$ concentrations in ZnO. Coexistence of ${\mathrm{Li}}_{i}^{+1}$ and ${\mathrm{Li}}_{\mathrm{Zn}}^{-1}$ at 1:2 and 2:1 ratios is marked by “×” and “+”, respectively. d) Migration barrier heights for Li‐ion migration paths in ZnO (see Figure S4 in the Supporting Information). e) Schematic of a thin‐film structure identical to the Li‐well electrode configuration of the LWOM device, consisting of Au (50 nm)/Li (5 nm)/ZnO (30 nm) on a SiO_2_ (300 nm)/p⁺‐Si substrate. f) SIMS profiles of the sample in panel (e) in its pristine state (as deposited) and after baking at 230 °C for 5 and 20 min. g) TEM dark‐field image of a cross section of the sample baked at 230 °C for 30 min.

**Figure** **1**a illustrates the electrical symbol and main concept of the LWOM. In ionic memristors, ion migration is driven by electric fields and Joule heating from current.^[^ ^20^ ^]^ The dominant driving force—electric field or Joule heating—determines switching behavior.^[^ ^20^, ^21^ ^]^ For instance, in TiO_2−*x*_, the initial resistance state (oxidation level) determines whether filament‐ or interface‐type switching occurs.^[^ ^22^, ^23^, ^24^ ^]^ The relative contribution of these forces varies with the memristor's conductance state, contributing to asymmetric weight update characteristics. In the LWOM, Li is locally injected at the source interface to form a Li well, with *V*_G_ and *V*_D_ controlling Li⁺‐ion migration. Under a fixed write *V*_D_ condition, the channel conductance varies with *V*_G_, altering the local electric field (voltage drop) at the electrode interface. Thus, the current through the memtransistor and the interfacial electric field, both functions of *V*_G_, enable precise control of weight update characteristics via *V*_D_ and *V*_G_ combinations.

## 2 Results and Discussion

### 2.1 ZnO Semiconductor and Li‐Well Fabrication

The wurtzite ZnO crystal exhibits an ABAB stacking of oxygen ions, with Zn ions occupying half of the tetrahedral sites (Figure 1b).^[^ ^25^ ^]^ The vacant octahedral sites offer suitable sites for Li‐ion incorporation and facilitate its migration. Li diffusion into ZnO can result in two primary defect types: substitutional Li (${\mathrm{Li}}_{\mathrm{Zn}}^{-1}$), acting as a p‐type defect, and interstitial Li (${\mathrm{Li}}_{i}^{+1}$), acting as an n‐type defect (Figure 1b).^[^ ^25^, ^26^, ^27^ ^]^ For simplicity, we consider only these two dominant defect types. To analyze electronic structure changes with Li doping position, density functional theory (DFT) calculations were performed on a supercell, varying Li concentration and site (Figure 1c). For ${\mathrm{Li}}_{i}^{+1}$, the conduction band (CB) shifts significantly below the Fermi level, exhibiting metallic n‐type behavior, with the conduction band minimum (CBM) decreasing by up to −4.3 eV and saturating above ≈5 at%. Conversely, ${\mathrm{Li}}_{\mathrm{Zn}}^{-1}$ shifts the CBM in the opposite direction, acting as a p‐type dopant. Projected band analysis reveals that the contribution of Li states increases in the CB for ${\mathrm{Li}}_{i}^{+1}$ and in the valence band (VB) for ${\mathrm{Li}}_{\mathrm{Zn}}^{-1}$ as the Li concentration rises (Figure S2, Supporting Information). When ${\mathrm{Li}}_{i}^{+1}$ and ${\mathrm{Li}}_{\mathrm{Zn}}^{-1}$ coexist, the CBM shift partially cancels out, resulting in either n‐ or p‐type characteristics depending on their relative ratio (Figure 1c; Figure S3, Supporting Information). These results quantitatively demonstrate that ZnO's electronic properties can be tailored by controlling Li doping type distribution.

Nudged elastic band (NEB) calculations of Li‐ion migration paths in ZnO reveal a high energy barrier of 2.4 eV for ${\mathrm{Li}}_{\mathrm{Zn}}^{-1}$ to ${\mathrm{Li}}_{i}^{+1}$ conversion due to strong bonding with oxygen, limiting ion mobility. In contrast, interstitial ${\mathrm{Li}}_{i}^{+1}$ migration exhibits significantly lower barriers: 0.69–0.90 eV in bulk and 0.14–1.40 eV along grain boundaries (GBs), depending on position and direction (Figure 1d; Figure S4, Supporting Information). Paths like GB1 and GB5, which exhibit wider diffusion pathways, show lower barriers, suggesting rapid Li‐ion transport via GBs. This mobility difference implies that, under an electric field, strongly bound ${\mathrm{Li}}_{\mathrm{Zn}}^{-1}$ remains fixed, while mobile ${\mathrm{Li}}_{i}^{+1}$ modulates the Schottky barrier near the interface.

To investigate Li diffusion, thin‐film samples mimicking the LWOM electrode structure (Figure 1e) were fabricated, and Li diffusion at 230 °C was analyzed via secondary ion mass spectrometry (SIMS) (Li melting point ≈180 °C). SIMS results for pristine samples and those baked at 230 °C for 5 and 20 min (Figure 1f) show increased Li diffusion into ZnO with longer bake times. Li diffuses into both Au and ZnO; however, its ionization efficiency in Au is low under Cs⁺ sputtering conditions (detectable with O_2_⁺, see Figure 3i). Within 5 min, Li reaches the SiO_2_ substrate, with increased accumulation at the SiO_2_ interface after 20 min. Notably, Li does not diffuse into amorphous SiO_2_ at this temperature, suggesting SiO_2_ (or Al_2_O_3_ and HfO_2_) could serve as a diffusion barrier in complementary metal–oxide–semiconductor (CMOS) processes.^[^ ^28^, ^29^ ^]^ Transmission electron microscopy (TEM) dark‐field images of a 20 min 230 °C baked sample (Figure 1g) show a dark region within ≈10 nm from the Au interface, followed by linear dark features extending further, indicating bulk, dislocation, and GB diffusion dominating up to 10 nm, with GB‐dominated diffusion beyond this depth. Li *K*‐edge electron energy loss spectroscopy (EELS) within 10 nm detected no Li 1s peak, suggesting <1 at% Li in ZnO, with most diffusing into Au (see Figure 3i).

### 2.2 Symmetric and Asymmetric Li‐Well Oxide Memtransistor

**Figure 2:**
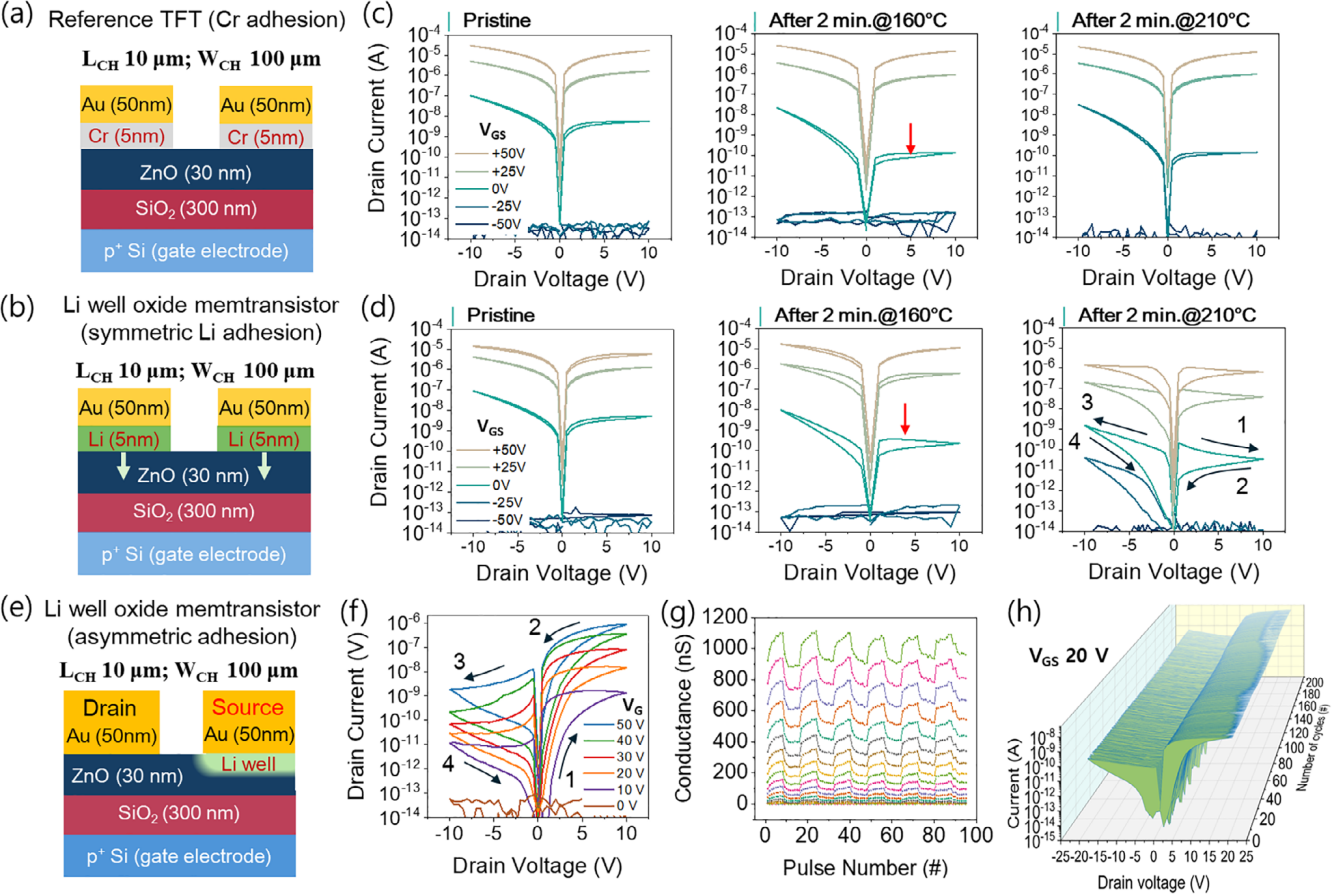
Comparison of a reference TFT with Cr adhesion and LWOM with Li adhesion. a) Cross‐sectional schematic of a reference TFT with Cr adhesion. b) Cross‐sectional schematic of a symmetric LWOM with Li adhesion. Both devices share identical structures (channel length 10 µm and channel width 100 µm). c) Output curves of the reference TFT in a pristine state, after 2 min bake at 160 °C, and after 2 min bake at 230 °C. d) Output curves of the symmetric LWOM in a pristine state, after 2 min bake at 160 °C, and after 2 min bake at 230 °C. Measured with *V*_DS_ 10 V sweeps at *V*_GS_ = −50, −25, 0, +25, and +50 V. Numbers and arrows in the 210 °C baked LWOM output curve indicate the sweep sequence. e) Cross‐sectional schematic of an asymmetric LWOM with a SiO_2_/p⁺‐Si back gate. f) Output curves of the asymmetric LWOM. Black arrows and numbers indicate the sweep sequence, showing counterclockwise switching. g) 8‐state (3 bit) long‐term potentiation/depression (LTP/LTD) characteristics plotted on a linear scale. Measured over six cycles (3‐bit round trip) with equal ±90 µA current conditions: potentiation at *V*_G_ = +50 V, *V*_DS_ = +5 V (100 µs); depression at *V*_G_ = +50 V and *V*_DS_ = −30 V (100 µs). h) Switching curve measured by 200 consecutive *V*_DS_ = 20 V sweeps at *V*_GS_ = 20 V.

**Figure** **2**a,b presents schematics of a reference TFT with a Cr adhesion layer and a symmetric LWOM with a Li adhesion layer, respectively. In the LWOM, a 5 nm thick lithium layer, used as an adhesion layer at the interface between the Au source/drain electrodes and the ZnO channel, serves a dual purpose: 1) acting as a doping source via thermal diffusion during the bake process and 2) facilitating electrode patterning during the lift‐off process. Figure 2c,d shows output curves for the reference TFT and symmetric LWOM under three conditions: pristine, after a 2 min heat treatment at 160 °C, and after a 2 min heat treatment at 210 °C (the measurements in Figure 2c,d were obtained from a single device subjected to incrementally increasing bake temperatures.). The output curves of the pristine reference TFT and LWOM devices are identical. However, after a 2 min bake at 160 °C, a small hysteresis loop appears in the LWOM output curve, becoming more pronounced after a 2 min bake at 210 °C. In contrast, the reference TFT with a Cr adhesion layer exhibits no hysteresis loop under any of the tested conditions. These results suggest that significant Li diffusion into ZnO begins at temperatures above 160 °C, with the extent of diffusion increasing at 210 °C, thereby amplifying the hysteresis loop (Figure 2d).

Output curves measured with increasing *V*_DS_ for devices baked at 210 °C (Figure S5a,b, Supporting Information) reveal that the hysteresis loop in the LWOM grows with higher *V*_DS_, whereas the reference TFT shows no hysteresis even at a *V*_DS_ sweep of 30 V. For both the reference TFT and LWOM, the current level at *V*_GS_ = 0 V decreases in baked devices compared to their pristine states (indicated by red arrows in Figure 2c,d). This reduction is attributed to decreased residual ligands and increased oxidation states in the ZnO channel layer following a 20 min bake at 230 °C (Figure S6, Supporting Information). Transfer curves measured at *V*_DS_ = 0.1 V before and after heat treatment for both the reference TFT and symmetric LWOM (Figure S7a,b, Supporting Information) demonstrate a uniform positive shift in turn‐on voltage due to increased oxidation. Intriguingly, despite Li injection at the interface post heat treatment, the LWOM exhibits turn‐on and threshold voltages identical to those of the reference TFT. This suggests that Li incorporation into ZnO forms a balanced ratio of ${\mathrm{Li}}_{\mathrm{Zn}}^{-1}$ and ${\mathrm{Li}}_{i}^{+1}$ defects (and/or additional oxygen supply), minimally altering the Fermi level near the ZnO interface.

The symmetric LWOM baked at 210 °C displays gate‐tunable memristive behavior with symmetric resistive switching characteristics, as indicated by black arrows (Figure 2d). During a positive *V*_DS_ sweep, the device switches from a low‐resistance state (LRS) to a high‐resistance state (HRS) (1 → 2); upon crossing 0 V into a negative voltage sweep (2 → 3), it starts in LRS and switches back to HRS (3 → 4). This symmetric switching behavior is typical of memristor devices where both electrode interfaces participate in resistive switching.^[^ ^1^, ^30^ ^]^ The migration direction of ${\mathrm{Li}}_{i}^{+1}$ ions is determined by the programming voltage. In the symmetric LWOM, where Li wells are formed beneath both source and drain electrodes, a *V*_DS_‐induced HRS switch at one electrode results in an LRS interface at the other.

To eliminate symmetric switching and impart directionality, we fabricated an asymmetric LWOM with a channel width of 100 µm and a length of 10 µm (Figure 2e). Here, the drain employs Cr adhesion (as in the reference TFT), while a Li well is formed only at the source region. Figure 2f–h presents the output curve, long‐term potentiation/depression (LTP/LTD) curves, and 200‐cycle resistive switching properties of the asymmetric LWOM, respectively. Asymmetric switching is observed, as indicated by the switching sequence (black arrows) in Figure 2f, driven by resistance changes at the source interface (switching mechanism discussed in the following section). This demonstrates control over on/off ratios and current levels via gate voltage. Figure 2g depicts 8‐state (3 bit) LTP/LTD characteristics in linear scale, measured at read voltages of *V*_DS_ = +1 V and *V*_GS_ ranging from +2.5 to +50 V (2.5 V steps). These results confirm that *V*_GS_ enables precise control of current levels and on/off ratios during read operations. Finally, Figure 2h shows consistent performance over 200 consecutive *V*_DS_ = 20 V sweeps at *V*_GS_ = +20 V, highlighting the device's uniformity.

### 2.3 Resistive Switching Mechanism of Li‐Well Oxide Memtransistor

**Figure 3:**
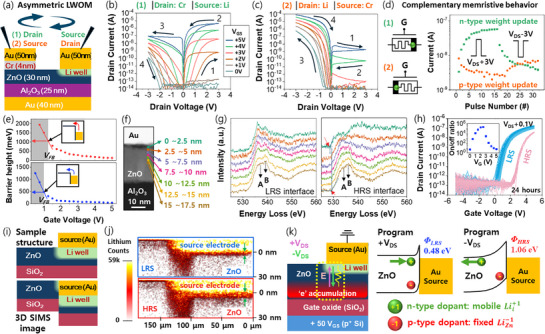
Resistive switching mechanism of the LWOM device. a) Cross‐sectional schematic of an asymmetric LWOM device. To impart directional resistive switching, one electrode uses diffusion‐free Cr adhesion, while the other employs Li adhesion, forming a Li well beneath one electrode via a 20 min bake at 230 °C. b) Output curve measured with the Cr adhesion electrode as drain and the Li‐well electrode as source. *V*_GS_ ranged from 0 to +5 V, with *V*_DS_ swept ±3 V at each *V*_GS_. c) Output curve measured with the Li‐well electrode as drain and the Cr adhesion electrode as source. (Panels (b) and (c) were obtained from the same device by swapping source–drain contacts.) d) 16‐state LTP/LTD curves measured by switching drain and source contacts: 1) Li well as the source: n‐type weight update (green curve); 2) Li well as the drain: p‐type weight update (orange curve). States 1–16 were updated with *V*_DS_ = +3 V (100 µs), *V*_GS_ = +5 V (LTP), followed by states 17–32 with *V*_DS_ = −3 V (100 µs), *V*_GS_ = +5 V (LTD). Read conditions were *V*_DS_ = +0.1 V, *V*_GS_ = +3 V; results for *V*_GS_ = +2–+5 V are shown in Figure S9 (Supporting Information). e) Schottky barrier height versus gate voltage for HRS and LRS measured on the same device. Arrows indicate barrier heights at flat‐band voltage. f) TEM dark‐field image of the source electrode Li‐well region. Arrows denote distances from the electrode interface edge. g) O *K*‐edge EELS spectra from LRS (left) and HRS (right) interfaces. Spectrum colors correspond to arrow and region colors in panel (f). h) Transfer curves (*V*_DS_ = +0.1 V) repeatedly measured over 24 h for HRS and LRS. Inset shows *I*_LRS_/*I*_HRS_ at various gate voltages. i) Schematic of the actual structure (top) and 3D SIMS‐derived image (bottom) of the LWOM analysis region. j) 3D SIMS results showing Li distribution in the Li‐well source electrode region for LRS (top) and HRS (bottom). An O_2_⁺ sputtering ion source maximized Li detection sensitivity, confirming that most Li from the 5 nm adhesion layer diffused into the Au electrode and was depleted. The depth scale was inferred from the Zn signal, and the *x*‐axis scale was estimated from the sputtered area. k) Device schematic (left) and band diagram (right) illustrating the switching mechanism during weight updates in the LWOM.

To enable low‐power operation, we designed and fabricated an asymmetric LWOM featuring an Al_2_O_3_ gate insulator (25 nm), a 4 µm channel length, and a 70 µm channel width (**Figure** **3**a; Figure S8, Supporting Information).To pre‐examine the resistive switching mechanism, we measured output curves and LTP/LTD curves by swapping source and drain contacts on the same device, with results presented in Figure 3b–d. Figure 3b shows the output curve with the Li‐well electrode as the source and the Au/Cr electrode as the drain, exhibiting counterclockwise switching behavior following the sweep sequence: LRS switching occurs at positive *V*_DS_ and HRS switching at negative *V*_DS_. Conversely, when the Li‐well electrode is set as the drain and the Au/Cr electrode as the source (Figure 3c), clockwise switching is observed, with a reduced hysteresis loop size. With the Li well at the source, a positive drain voltage pushes Li⁺ cations toward the source interface, lowering the Schottky barrier, while a negative voltage pulls them away, increasing the barrier. When the Li well is the drain, a positive voltage repels Li⁺ cations from the drain interface, forming a high Schottky barrier and inducing HRS switching. Since the drain interface operates in forward bias, its current variation is smaller compared to the reverse‐biased Schottky barrier modulation at the source Li well. Additionally, the smaller potential difference between the drain and gate during positive *V*_DS_ sweeps likely reduces ${\mathrm{Li}}_{i}^{+1}$ migration. A stronger local electric field at the reverse‐biased source interface under positive *V*_DS_ enhances Li⁺ migration, resulting in a higher on/off ratio when the Li well is at the source. This trend is mirrored in the LTP/LTD curves (Figure 3d), where the Li well as the source (green curve) shows potentiation with positive pulses and depression with negative pulses, while the Li well as the drain (orange curve) exhibits the reverse behavior. To distinguish these characteristics, we denote conductance increases with positive voltage as n‐type weight updates and decreases with negative voltage as p‐type weight updates (see Figure S9 in the Supporting Information for *V*_GS_‐dependent n‐type and p‐type characteristics).

To verify interface resistance changes, we recorded transfer curves for HRS and LRS states over a temperature range of 305–350 K in 5 K increments, extracting barrier height values at each *V*_GS_ from ln (*I*_D_/*T* ^2^) versus temperature plots. Using flat‐band voltage extrapolation, we determined the barrier height to be ≈1067 meV for HRS and 479 meV for LRS, indicating an ≈588 meV shift during LRS‐to‐HRS switching (Figure 3e). For detailed analysis, we performed O *K*‐edge EELS on cross sections of HRS and LRS devices, with results shown in Figure 3f,g (measured ≈80 nm from the source electrode edge). Figure 3f displays a TEM dark‐field image of the source Li‐well region. Figure 3g shows O *K*‐edge EELS spectra obtained by summing data over 2.5 nm depth intervals from 0 to 17.5 nm, color‐coded to correspond to regions in Figure 3f. Beyond 13 nm from the interface, both LRS and HRS exhibit typical ZnO O *K*‐edge spectra. Peaks A and B, representing density of states (DOS) from O 2p and Zn 4s orbital mixing, correspond to σ‐bonding along the *c*‐axis (on‐axis, peak A) and π‐bonding in the *a–b* plane (off‐axis, peak B), respectively. In LRS, peak B intensity decreases relative to peak A within 12 nm of the interface, becoming more pronounced closer to the Li‐rich boundary. DFT calculations reveal that excess electrons introduced by n‐type Li interstitial doping primarily redistribute into *a–b* plane Zn─O bonds, preferentially occupying unoccupied O p*_x_*/p*_y_*‐derived states (Figure S10, Supporting Information). Crystal orbital Hamiltonian population (COHP) analysis confirms that increasing Li doping reduces unoccupied Zn─O bonding states, more significantly in the p*_x_*/p*_y_* directions than p*_z_*, directly explaining the systematic reduction in peak B intensity observed in EELS spectra. In HRS, peak B intensity decreases relative to peak A only between 5 and 7.5 nm, with both peaks diminishing closer to the interface, likely due to structural distortion or disorder from ${\mathrm{Li}}_{i}^{+1}$ dispersion, leaving ${\mathrm{Li}}_{\mathrm{Zn}}^{-1}$. Without energy calibration, EELS data reveal an ≈2.5 eV shift between LRS and HRS samples. In LRS, the O *K*‐edge energy remains unchanged from 15–17.5 to 0–2.5 nm, whereas in HRS, an ≈0.6 eV redshift occurs at 0–2.5 nm compared to 15–17.5 nm (marked by red arrows, edge energy defined by extrapolation). Assuming this shift reflects a redshift in O 1s binding energy, it suggests an ≈0.6 eV Fermi level shift toward the valence band maximum due to local Li concentration changes,^[^ ^31^ ^]^ consistent with the Schottky barrier increase derived from flat‐band voltage. This barrier height increase shifts the turn‐on voltage in transfer curves (Figure S8b, Supporting Information), with no hysteresis observed in *V*_GS_ sweeps, indicating the absence of defect‐related charge trapping. Figure 3h shows transfer curves measured every 16 min over 24 h for LRS and HRS, confirming stable state retention. The inset in Figure 3h illustrates that the on/off ratio decreases with increasing *V*_GS_, as the Schottky barrier difference becomes negligible at higher gate voltages.

For clearer mechanistic insights, 3D SIMS analysis was conducted. Figure 3i depicts the sample structure used (top) and the detected structure from 3D SIMS data (bottom). Due to SIMS sputtering from the surface, the height difference between the ZnO channel and source electrode should be considered in the 3D SIMS image. Figure 3j shows 3D SIMS images of Li‐ion distribution at the source electrode region for LRS (top) and HRS (bottom). In HRS, Li distribution spreads more vertically from the Au electrode compared to LRS, rather than along the drain direction. The image base corresponds to the gate oxide interface, with ${\mathrm{Li}}_{i}^{+1}$ migration primarily at the electrode‐channel edge, though distribution changes are also observed inland (marked by green arrows). The switching mechanism is illustrated in Figure 3k with a device schematic (left) and band diagram (right). Applying +*V*_GS_ accumulates electrons at the gate oxide interface, forming a channel. With +*V*_DS_, the region between the source electrode and channel beneath it exhibits the highest resistance in the drain‐channel‐source electrical path (yellow resistor in Figure 3k), resulting in a strong local electric field (purple in Figure 3k). This field drives ${\mathrm{Li}}_{i}^{+1}$ migration, inducing Fermi level changes. Upon application of *V*_DS_, the depletion width of the Li:ZnO/Au interface Schottky barrier, which acts as the dominant resistance in the drain‐channel‐source current path, decreases in proportion to the electric field applied by +*V*_GS_. Consequently, the write voltage *V*_DS_ results in a strong electric field concentrated in the reduced depletion width, inducing Li migration. This suggests that the promotion and attenuation of Li migration can be controlled by *V*_GS_. Thus, reversible Li‐ion movement in this local region enables low‐voltage write operations despite the 4 µm channel length, suggesting that switching voltage depends more on channel charge induced by the gate and channel thickness than length. During baking, Li occupies Zn and interstitial sites in ZnO at a specific ratio. As shown in the Figure 3k band diagram, ${\mathrm{Li}}_{\mathrm{Zn}}^{-1}$ is strongly bound and acts as a fixed p‐type dopant, while ${\mathrm{Li}}_{i}^{+1}$, more mobile under the local electric field during programming, departs from the source interface, raising the Schottky barrier via remaining ${\mathrm{Li}}_{\mathrm{Zn}}^{-1}$. Conversely, −*V*_DS_ pushes ${\mathrm{Li}}_{i}^{+1}$ back to the source interface, lowering the barrier and shifting turn‐on *V*_GS_ to 0 V.

### 2.4 Memristive Properties of Li‐Well Oxide Memtransistor

**Figure 4:**
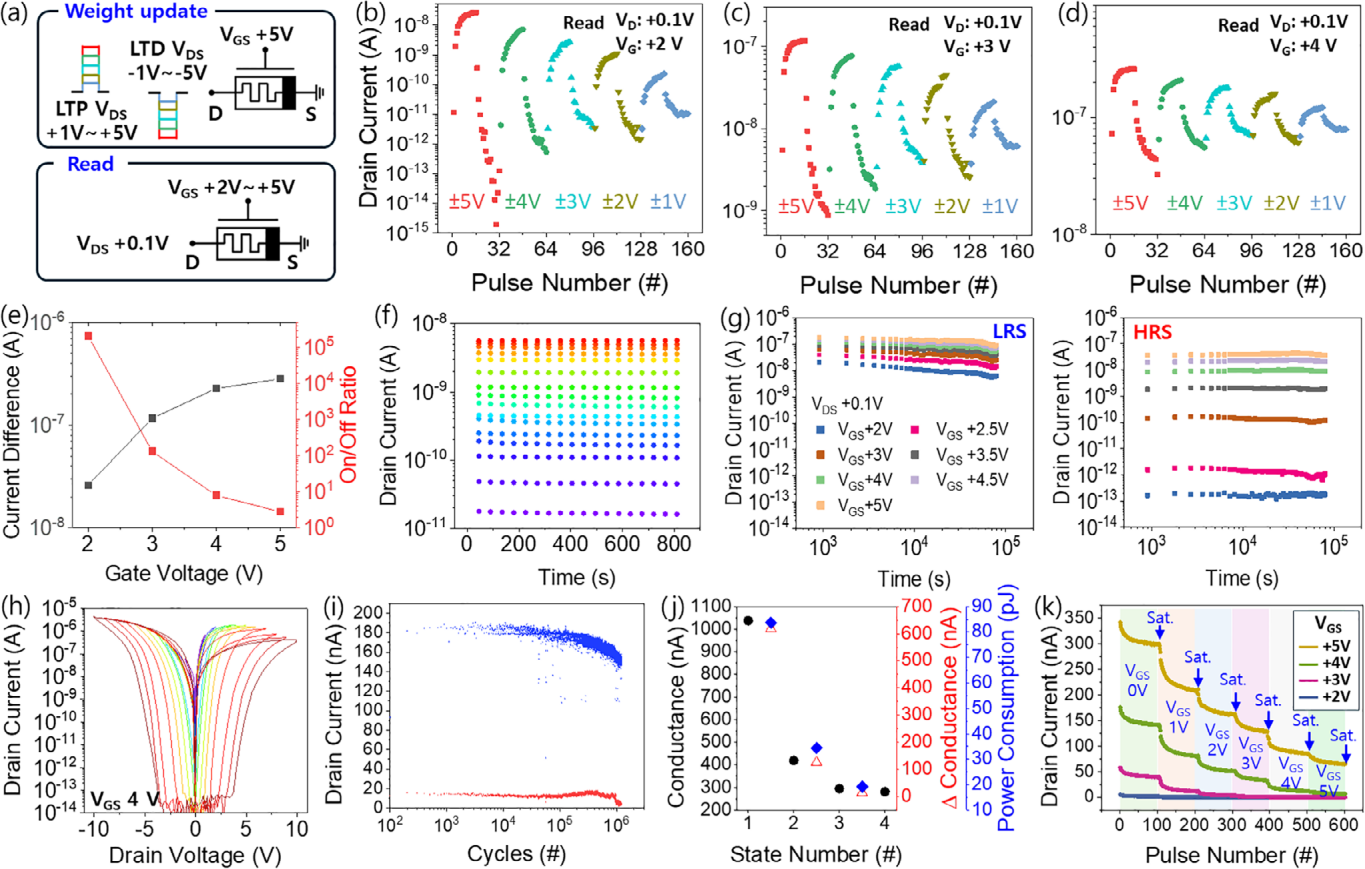
Memristive characteristics of the LWOM device. a) Schematic illustrating voltage conditions for weight update (top) and read (bottom) operations. b–d) 16‐state LTP/LTD curves obtained by weight updates with *V*_GS_ = +5 V and *V*_DS_ = 1–5 V (100 µs), followed by reads at *V*_DS_ = +0.1 V with *V*_GS_ = +2, +3, and +4 V, respectively. e) On–off current difference (left axis) and on/off ratio (right axis) as a function of read gate voltage. f) Retention characteristics of each of the 16 states. Weight updates were performed with *V*_GS_ = +5 V, *V*_DS_ = +3 V (100 µs), and read at *V*_DS_ = +0.1 V, *V*_GS_ = +2 V. Retention data for *V*_GS_ = +3, +4, and +5 V are shown in Figure S13 (Supporting Information. g) Retention characteristics measured over 24 h for full LRS (left) and full HRS (right), plotted for *V*_GS_ from +2 to +5 V in 0.5 V increments. h) DC sweep curves showing *V*_DS_ sweeps from 1 to 10 V at *V*_GS_ = +4 V. i) Endurance measured up to 1.2 × 10⁶ cycles using *V*_DS_ pulses (1 ms, ±5 V) and *V*_GS_ pulses (1 ms, +3 V) for switching, with read operations performed at *V*_DS_ = +0.1 V and *V*_GS_ = +3 V. j) Conductance values (black) obtained from DC reads at *V*_DS_ = +0.1 V and *V*_GS_ ​ = +2 V for four representative states, the corresponding conductance changes between states (Δ*G*, red), and the calculated power consumption for each weight update transition (blue). k) Saturation curves of weight updates as a function of *V*_GS_ from the weight adaptation test.

**Figure** **4**a illustrates the weight update measurement method using *V*_DS_ pulses with a 100 µs width and amplitudes ranging from ±1 to ±5 V, all with *V*_GS_ = +5 V. Four 16‐state LTP/LTD curves, shown in Figure 4b–d and Figure S11 (Supporting Information), were read at *V*_DS_ = +0.1 V with *V*_GS_ values of +2, +3, +4, and +5 V, respectively. Each figure presents four weight update curves corresponding to different *V*_DS_ pulses (from ±1 to ±5 V, 100 µs). The magnitude of weight updates increases proportionally with *V*_DS_ amplitude and decreases as the read *V*_GS_ increases, indicating that higher *V*_DS_ enhances Li‐ion migration, while *V*_GS_ partially compensates for Schottky barrier differences. The on/off ratio and current differences observed in Figure 4b–d (and Figure S11, Supporting Information) are summarized in Figure 4e (using maximum and minimum currents from weight update curves). The on/off ratio decreases with increasing read *V*_GS_, while the current difference between states increases, enabling flexible operation based on circuit requirements for current differentiation and power consumption. Retention tests conducted over 810 s at 45 s intervals confirm stable conductance across 16 states (Figure 4f). Figure 4g presents the retention characteristics of fully switched LRS and HRS states by *V*_GS_ = +5 V and *V*_DS_ = +3 V, confirming stable data retention over 24 h. Figure 4h presents DC sweep curves from *V*_DS_ = 1–10 V at *V*_GS_ = +4 V, with on/off ratio and switching voltage increasing with sweep amplitude, proportional to Li‐ion migration (Figure S13, Supporting Information). Figure 4i presents pulse‐switching endurance data measured up to 1.2 × 10⁶ cycles. To ensure a clear distinction between the ON and OFF states, 1 ms pulses were applied, and to shorten the overall measurement time, a read operation was performed once every 100 pulse‐switching cycles. (For reference, the endurance result obtained with a read after every switching event for 1.1 × 10⁴ cycles is shown in Figure S14 in the Supporting Information.) No degradation in the on/off ratio was observed up to 1.2 × 10⁶ cycles; however, a slight decrease in current for both states was noted as the number of switching cycles increased. (In Figure S14 in the Supporting Information, this behavior was not observed in a separate device tested up to 10⁴ cycles.) This gradual shift is attributed to the difference in power required for HRS and LRS switching, which causes the state to progressively move toward one side under symmetric switching pulse conditions. In addition, the HRS, having a lower current level and being more sensitive to state variations, exhibited larger fluctuations compared to the LRS. To evaluate the power consumption during weight update in the LWOM device, conductance was monitored via DC read at *V*_DS_ ​ = +0.1 V and *V*_GS_ = +2 V, while weight updates were performed using pulses with a width of 100 µs and amplitudes of *V*_GS_ = +2 V and *V*_DS_ ​ = −2 V. The current measured during each weight update pulse is shown in Figure S15 (Supporting Information), from which the corresponding power consumption was calculated. In Figure 4j, the black plot represents the conductance values measured from DC reads for four weight states (#1: 1038.01 nS, #2: 419.98 nS, #3: 294.70 nS, and #4: 281.54 nS), while the red plot depicts the change in conductance (Δ*G*) between successive states (#1 − #2: 618.03 nS, #2 − #3: 125.28 nS, and #3 − #4: 13.24 nS). As shown by the blue plot, the corresponding power consumptions for these transitions are 83.66, 34.42, and 19.26 pJ, respectively, yielding Δ*G*‐to‐power ratios of 7.39, 3.64, and 0.68 nS pJ^−1^. Notably, lower conductance states exhibited reduced power consumption during weight updates. While in typical two‐terminal devices a lower conductance reduces the write power at the expense of longer read times, this trade‐off can be mitigated in three‐terminal devices by amplifying the read current through gate control. Based on the LWOM device characteristics measured thus far, the performance of three types of memtransistor devices (ion migration, ferroelectric, and charge trap) was compared and presented in Table S1 (Supporting Information). For charge‐trap‐type memtransistors, the write voltage (and write speed) depends on the tunnel oxide thickness, resulting in a trade‐off between the write voltage magnitude and retention characteristics. Consequently, there is a limitation in reducing the write voltage while ensuring robust retention. FeFETs offer advantages in writing voltage (3–4 V) and weight update speed (from 40 ns to 1 µs); however, they face technical challenges related to retention degradation caused by depolarization fields and charge trapping, along with the characteristic of performing write operations using only *V*_GS_ (which poses a burden due to crosstalk issues in crossbar array configurations). Ionic migration‐type memtransistors, on the other hand, require substantial energy for ion migration and exhibit slow speeds, making them less competitive compared to existing FeFETs and charge‐trap‐type memtransistors, as indicated in Table S1 (Supporting Information). The present LWOM device exhibits a significantly reduced operating voltage (<3 V) and an improved speed (≈100 µs) compared to conventional ionic migration‐type memtransistors. Nevertheless, due to its reliance on ion migration, the write speed remains slower than that of FeFETs, which exploit ultrafast ferroelectric polarization switching, or charge‐trap devices, which utilize rapid electron trapping/detrapping. This intrinsic limitation represents a key challenge for the LWOM. Based on the insights gained in this work, we anticipate that further optimization—such as employing channel materials with higher ionic mobility, integrating high‐*k* dielectric layers to enhance electrostatic control, and scaling down the device dimensions—could substantially improve the programming speed. Additionally, from a long‐term endurance perspective, potential risks of Li segregation and redistribution should be considered. However, the standard reduction potential (SRP) of Li (−3.04 V) is far more negative than those of Cu (+0.34 V) and Ag (+0.80 V) which readily form metallic filaments in oxides.^[^ ^32^, ^33^, ^34^ ^]^ This large difference implies that, unlike Cu⁺ or Ag⁺ ions, Li⁺ ions are relatively unlikely to segregate into metallic Li within oxides. Consequently, redistribution and diffusion present a more critical concern than segregation. To mitigate these effects, the development of high‐quality diffusion barriers (such as dense oxides or nitrides) will be essential to suppress Li‐ion migration during prolonged cycling.^[^ ^35^, ^36^, ^37^ ^]^

To control the saturation point of weight updates, we conducted a weight adaptation test. In the LWOM, *V*_GS_ determines electron accumulation in the channel, setting conductance and the local electric field at the interface (channel and interface resistances in series). Repeated *V*_DS_ pulses at a given *V*_GS_ redistribute Li ions, altering interface resistance until a saturation point is reached where channel and interface resistances balance. Thus, saturation points can be tuned by *V*_GS_. As shown in Figure S16 (Supporting Information), we performed the test by incrementing *V*_GS_ from 0 to 5 V in 1 V steps, applying 100 *V*_DS_ = −2 V (1 ms) pulses per step to observe saturation, then increasing *V*_GS_ and repeating (results in Figure 4k). This confirms *V*_GS_‐controlled saturation, implying a conductance change region operable without inhibit voltages in crossbar arrays, enabling operation without dual gates.^[^ ^38^ ^]^ In the crossbar array architecture used in this study, memtransistor devices in each row share drain and source electrodes (Figure S1b, Supporting Information). Consequently, when the weight update voltage is applied to the drain of a selected cell, data loss occurs in unselected cells. Therefore, a second gate is typically used to apply a −*V*_2G_ voltage (inhibit voltage) to unselected cells, completely blocking current to protect data. When a *V*_DS_ ​ pulse for weight updating is applied, the conductance saturates at a certain level beyond which no further update occurs. This property can be exploited to enable selective programming of individual devices in a crossbar array (Figure S1b, Supporting Information) without write disturbance. Figure S17a (Supporting Information) shows the saturation curves for LTP (from full HRS) and LTD (from full LRS), obtained by applying 100 pulses (width: 100 µs, *V*_GS_ = 0 V, and *V*_DS_ = ±2 V). The operational conductance window could be defined between the two saturation values. Figure S17b (Supporting Information) presents the results of a disturbance test during programming. The black plot shows the preset conductance values of devices #1 − #21 in a 21‐device parallel configuration. The red plot shows the conductance values after performing 17 LTD update pulses (width: 100 µs, *V*_GS_ ​ = +2 V, and *V*_DS_ ​ = −2 V) on device #21, and the blue plot shows the conductance values after performing 72 LTD update pulses (width: 100 µs, *V*_GS_ ​ = +2 V, and *V*_DS_ ​ = +2 V) on the same device. As evident in the figure, by utilizing the conductance range within the *V*_GS_ ​ = 0 V saturation region, individual devices can be programmed without the need for inhibit biases.

### 2.5 Li‐Well ZnO Memtransistor‐Based 21 × 21 Crossbar Array

**Figure 5:**
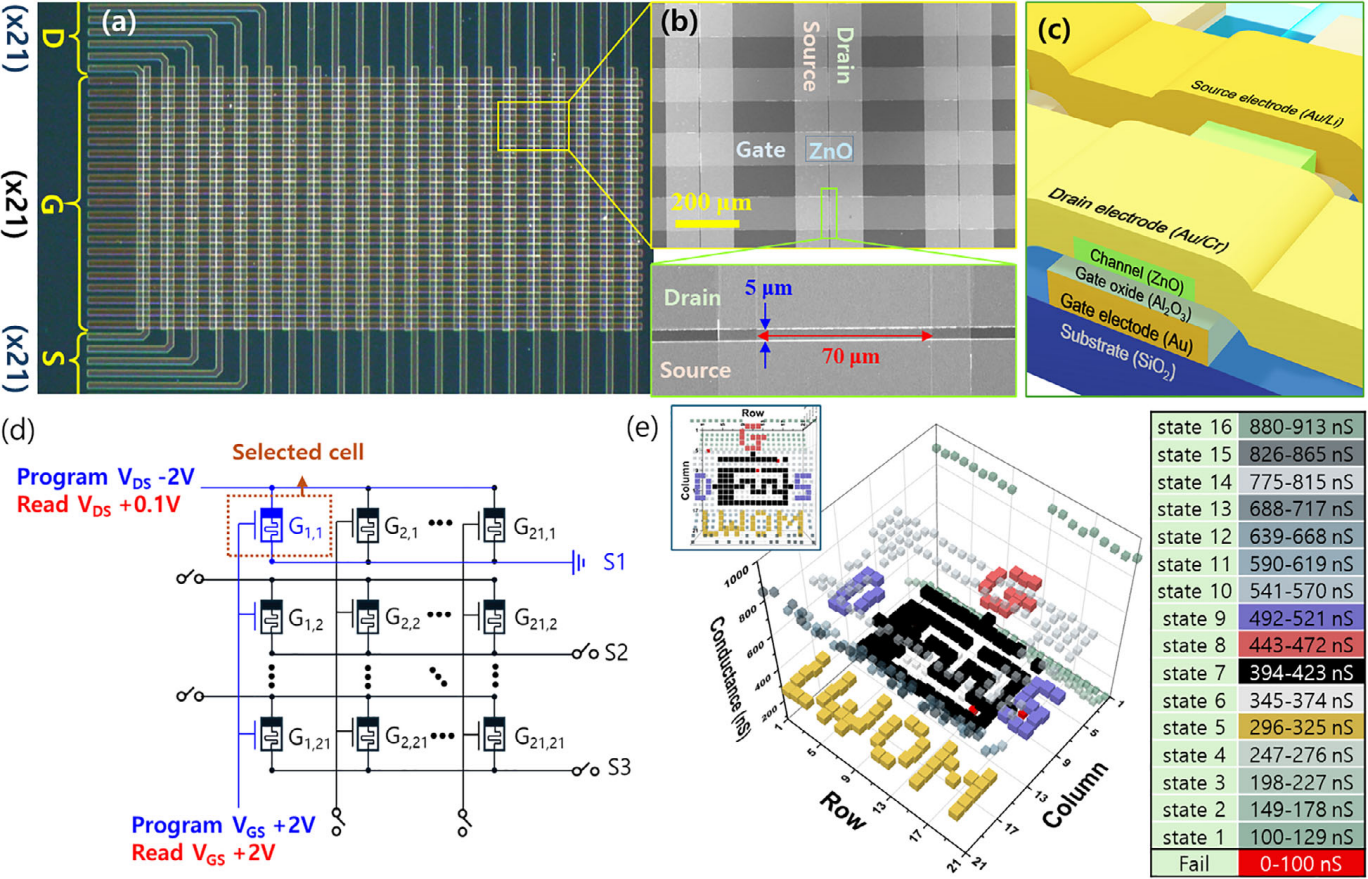
21 × 21 LWOM crossbar array. a) Optical photograph of the 21 × 21 LWOM crossbar array device. D, G, and S denote drain, gate, and source electrode lines, respectively, each comprising 21 electrode lines. Captured using the optical camera of a probe station; refer to the scale bar in panel (b) for scale. b) SEM image of a 4 × 3 array region (top) and a magnified image of a single device (bottom). The magnified region is marked by a box of the same color in the image. All LWOM devices in the 21 × 21 array, as shown in the magnified SEM image, were fabricated with a channel length of 5 µm and a channel width of 70 µm. c) Device schematic showing a cross section along the drain electrode. 100 µm width Au (35 nm)/Cr (4 nm) gate electrode line is coated with a 25 nm Al_2_O_3_ gate insulator, followed by a 70 µm width ZnO (30 nm) channel layer, and crossed by 100 µm width source Au (60 nm)/Li (5 nm) and drain Au (60 nm)/Cr (5 nm) electrodes. d) Circuit diagram illustrating the selection and operation of individual cells in the 21 × 21 LWOM crossbar array. e) 3D plot of conductance distribution across LWOM devices in the 21 × 21 array after training (left) and a table listing target conductance values and corresponding colors for each of the 16 states (right). (Inset at top left shows the 3D plot viewed along the *z*‐axis.).

To verify crossbar array functionality, we fabricated a 21 × 21 LWOM array with a simple structure (**Figure** **5**a; Figure S1b, Supporting Information). Each row's 21 LWOMs share source/drain electrodes, and each column's 21 LWOMs share gates. Figure 5b shows a scanning electron microscopy (SEM) image of a 4 × 3 array region (top) and a magnified single device (bottom). To provide structural insight, Figure 5c depicts a schematic cross section. Figure 5d illustrates the 21 × 21 array circuit, using gate, source, and drain electrodes to select cells for weight updates and reads. The devices sharing source/drain electrodes may lose data by the writing operation of neighboring cells. To demonstrate individual weight updates without the crosstalk, we set 16 conductance states below the *V*_GS_ = 0 V saturation point observed in Figure 4k (post‐baked initial state is LRS), performing depression updates with *V*_DS_ = −2 V (100 µs) and *V*_GS_ = +2 V until target conductance, read at *V*_DS_ = +0.1 V and *V*_GS_ = +2 V. Conductance ranges for the 16 states are listed in Figure 5e (right table). Assigning unique target states to each cell, we measured all cells’ conductance post update in a 3D plot (16 states in distinct colors). Out of 441 devices, only three devices failed, resulting in an exceptionally high operational yield of 99.31%. These failed devices exhibited no drain currents, suggesting that the failure was caused by channel layer damage during fabrication rather than being stuck in a weight update. Device‐to‐device variation affects update speed, but 438 devices successfully reached the target conductance within 91 pulses. Conductance distribution versus pulse count is provided in Video S1 (Supporting Information).

## 3 Conclusion

This study introduces a low‐power LWOM by forming a Li‐well beneath the source electrode in a ZnO TFT, reporting its performance and memristive switching mechanism. Its simple fabrication, 230 °C thermal budget, and use of mature oxide TFT technology suggest high scalability for neuromorphic hardware and next‐generation nonvolatile memory. This approach can be extended to other oxide semiconductors, offering the potential for enhanced LWOM performance, while vertical LWOM development using atomic layer deposition (ALD) and oxide TFT technologies appears feasible. High‐quality diffusion barriers to prevent Li diffusion will be crucial. The LWOM's superior memristive switching characteristics position it as a strong candidate alongside CTF and FeFET devices, and we anticipate this work will inspire further Li‐well memtransistor advancements.

## 4 Experimental Section

### Local‐Gate Asymmetric LWOM Device Fabrication

A 1.5 cm × 1.7 cm SiO_2_ (300 nm)/p‐Si substrate was ultrasonically cleaned with acetone, deionized (DI) water, and ethanol for 1 min each. All patterning steps were performed via photolithography using a mask aligner (M150S, Prowin). AZ GXR‐601‐14CP photoresist was spin‐coated at 4000 rpm for 60 s, followed by prebaking at 90 °C and postbaking at 110 °C. Patterns were exposed to 350 W UV light for 1.5 s and developed in AZ 300 MIF developer for 1 min, then rinsed with DI water. A 4 nm Cr adhesion layer and a 35 nm Au gate electrode were deposited using a thermal evaporator (SN‐TECH) at a base pressure of 6.0 × 10^−^⁷ Torr, with deposition rates of 0.8 Å s^−1^ for Cr and 1 Å s^−1^ for Au. The gate electrode was completed via lift‐off in acetone at 50 °C (Figure S18a, Supporting Information). A 25 nm Al_2_O_3_ gate insulator (200 cycles) was deposited by ALD (IC‐100, ITECHU) at 110 °C, using trimethylaluminum (TMA, Ezchem) as the aluminum precursor, DI water as the reactant, and high‐purity Ar (99.9999%) as the carrier gas. The ALD chamber maintained a base pressure of 0.375 Torr with a 50 sccm Ar flow. Each cycle consisted of TMA (1 s pulse), Ar (20 s purge), H_2_O (1 s pulse), and Ar (20 s purge), achieving a growth per cycle (GPC) of ≈1.25 Å (Figures S18b and S19a, Supporting Information). A 30 nm ZnO channel layer (200 cycles) was then deposited by ALD using diethylzinc (DEZ, Ezchem) as the zinc precursor, with a sequence of DEZ (0.5 s pulse), Ar (10 s purge), H_2_O (0.5 s pulse), and Ar (15 s purge), yielding a GPC of 1.5 Å at 110 °C (Figures S18c and S19b, Supporting Information). The ZnO channel was patterned via photolithography and wet‐etched with 0.1% HCl aqueous solution for 1 min, followed by photoresist removal with acetone (Figure S18d, Supporting Information). Drain electrodes were patterned by photolithography, and a 5 nm Cr adhesion layer and 50 nm Au were deposited via thermal evaporation, followed by lift‐off (Figure S18e, Supporting Information). Source electrodes were similarly patterned, with a 5 nm Li adhesion layer and 50 nm Au deposited at rates of 0.6 Å s^−1^ for Li and 1 Å s^−1^ for Au, completed by lift‐off. The resulting asymmetric LWOM had a channel length of 4 µm and a width of 70 µm (Figure S18f, Supporting Information).

### 21 × 21 Crossbar Array Fabrication

On a cleaned 2 cm × 1.5 cm SiO_2_ (300 nm)/p‐Si substrate, 21 gate electrodes were patterned via photolithography. A 4 nm Cr adhesion layer and 35 nm Au were deposited by thermal evaporation, followed by lift‐off (Figure S20a, Supporting Information). A 25 nm Al_2_O_3_ gate insulator (200 cycles) and a 30 nm ZnO channel layer (200 cycles) were deposited via ALD (Figure S20b,c, Supporting Information). The ZnO layer was patterned by photolithography and wet‐etched to define 441 individual channels, with residual photoresist being removed using acetone (Figure S20d, Supporting Information). Twenty‐one drain electrodes were patterned, and a 5 nm Cr adhesion layer and 50 nm Au were deposited, followed by lift‐off (Figure S20e, Supporting Information). Source electrodes were patterned, and a 5 nm Li adhesion layer and 50 nm Au were deposited, completed by lift‐off. The final 21 × 21 crossbar array had a channel length of 5 µm and a width of 70 µm (Figure S20f, Supporting Information).

### *I–V* Measurements

Electrical measurements were performed using a source measure unit (SMU, Keithley 2634B), an arbitrary function generator (AFG31152, Tektronix), a pulse function arbitrary noise generator (81150A, Keysight), a mixed‐domain oscilloscope (MDO343‐BW‐350, Tektronix), a customized switch matrix (SNM), and a 64‐pin multicontact probe card (MS‐TECH). The single‐device measurement sequence is shown in Figure S21a (Supporting Information). Signals were conditioned by the function generator and oscilloscope for desired amplitude and pulse width, routed through an RF switch. The gate terminal was directly connected to the source meter, bypassing the radio frequency (RF) switch. A gate voltage was applied first via the source meter, followed by drain pulses. Write operations (AC pulses) followed the sequence: AFG → RF switch → device; read operations (DC voltage) followed: SMU → RF switch → device → RF switch → SMU. For the 21 × 21 crossbar array, a switch matrix integrating 63 electrodes (21 sources, 21 drains, and 21 gates) enabled random‐access measurements (Figure S21b, Supporting Information). Equipment for array measurements is shown in Figure S22 (Supporting Information).

### TEM

Cross‐sectional TEM, scanning transmission electron microscope (STEM), and EELS analyses were conducted using a focused ion beam (FIB, Nova Nanolab, FEI) and high‐resolution TEM (HR‐TEM, JEM‐2100F, JEOL). A 700 nm carbon protective layer was deposited before FIB milling at 30 kV with a 7 nm beam width. A single asymmetric LWOM sample (10 µm lateral and 100 nm thick) was analyzed. HR‐TEM imaging used a 200 keV acceleration voltage, achieving ≈0.23 nm resolution. EELS data were collected with a 0.05 s exposure per point.

### SIMS Measurement

Au (50 nm)/Li (5 nm)/ZnO (30 nm) films were deposited on a SiO_2_ (300 nm)/p‐Si substrate via thermal evaporation and ALD, baked at 230 °C for 0, 5, and 20 min. Depth profiling used time‐of‐flight SIMS (TOF‐SIMS‐5, ION‐TOF) with a Cs⁺ ion gun over a 300 µm × 300 µm region.

### 3D SIMS Measurement

After programming two asymmetric LWOMs into LRS and HRS, 3D depth profiling was performed using TOF‐SIMS over a 150 µm × 150 µm region at the ZnO channel–Au/Li source interface, with an O_2_⁺ ion gun for enhanced Li detection.

### X‐Ray Diffraction Measurement

An Au (10 nm)/Li (6 nm)/ZnO (30 nm) film was deposited on a SiO_2_ (300 nm)/p‐Si substrate via thermal evaporation and ALD, annealed at 230 °C for 20 min. Crystal structure was analyzed by X‐ray diffraction (XRD, Dmax2500/PC, Rigaku) with Cu Kα radiation. Grazing incidence XRD (GIXRD) was performed at a 1° incidence angle, scanning 25°–80° in 0.04° steps with 1 s per step. Results, *d*‐spacing, and grain sizes (Scherrer equation) are provided in Figure S23 and TableS2 (Supporting Information).

### X‐Ray Photoelectron Spectroscopy Measurement

ZnO (30 nm) films were deposited on a SiO_2_ (300 nm)/p‐Si substrate via ALD. One sample was baked at 230 °C for 20 min; another remained pristine. X‐ray photoelectron spectroscopy (XPS, K‐Alpha, Thermo Electron) with an Al Kα source analyzed C 1s, O 1s, and Zn 2p core levels. Narrow scans (15 repeats) used a 50 eV pass energy and 0.1 eV steps, with a 400 µm X‐ray spot size on carbon‐taped samples.

### Computational Details

The first‐principles calculations were performed using the Vienna Ab initio Simulation Package (VASP)^[^ ^39^ ^]^ within the framework of DFT. The projector‐augmented wave (PAW) method^[^ ^40^ ^]^ was used to describe the electron‐ion interactions, and the generalized gradient approximation (GGA) in the Perdew–Burke–Ernzerhof (PBE) formulation was employed for the exchange‐correlation functional.^[^ ^41^ ^]^ To investigate the Li‐doping effects in ZnO, the DFT+*U* method was employed to account for the strong electron correlation effects, with an effective *U* value of 7.5 eV applied to the Zn 3d states.^[^ ^42^ ^]^ The energy cutoff for the plane‐wave basis set was set to 550 eV. Geometry optimizations were performed until the atomic forces were less than 0.01 eV Å^−1^ and the total energy convergence was below 10^−5^ eV. The migration barriers for Li migration were calculated using the climbing image NEB (CI‐NEB) method.^[^ ^43^ ^]^ The grain boundary structures were constructed using the Aimsgb code.^[^ ^44^ ^]^

## Conflict of Interest

The authors declare no conflict of interest.

## Supporting information



Supporting Information

Supplemental Movie 1

## Data Availability

The data that support the findings of this study are available from the corresponding author upon reasonable request.
