# Supplementary material for: Li‐Well ZnO Memtransistors: High Reliability for Neuromorphic Applications
Source: Adv Mater. 2025 Sep 10;38(1):e06128. doi: 10.1002/adma.202506128 (PMC12759231; doi:10.1002/adma.202506128)
Supplement: Supplementary file 1 — Supporting Information [file ADMA-38-e06128-s002.docx]

Supporting Information

**Li-Well ZnO Memtransistors: High Reliability for Neuromorphic Applications**

*Ki-Hoon Son,^a,#^ Hyun-Sik Kim,^a,#^ Dae-Hee Han,^a^ Hyung‐Kyu Lim,^b*^ Hong-Sub Lee^a*^*

Ki-Hoon Son, Hyun-Sik Kim, Dae-Hee Han, Prof. Hong-Sub Lee

^a^Department of Materials Science & Engineering, Kyung Hee University, Yongin 17104, Republic of Korea

E-mail: [h.s.lee@khu.ac.kr](mailto:h.s.lee@khu.ac.kr)

Prof. Hyung-Kyu Lim

^b^Department of Chemical Engineering, Interdisciplinary Program in Advanced Functional Materials and Devices Development, Kangwon National University, 1 Kangwondaehak-gil, Chuncheon, Gangwon, 24341 Korea

E-mail: [hklim@kangwon.ac.kr](mailto:hklim@kangwon.ac.kr)

^#^These authors contributed equally.

| 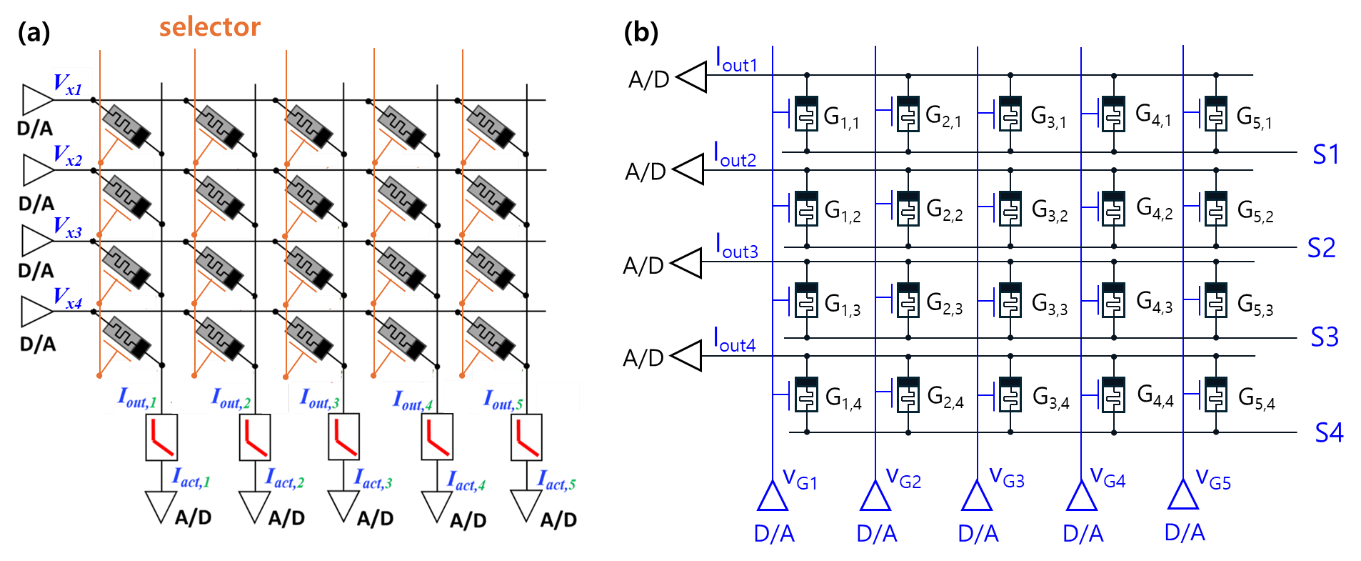 |
| --- |
| **Figure S1. Schematic of a crossbar array memtransistor-Based Vector-Matrix Multiplier Circuit.** a) Structure using the gate terminal as a selector and for current control. Analogue input data is applied as voltage to the drain for MAC operations. b) Structure using the drain terminal as a selector and for current control. Analogue input data is applied as voltage to the gate, with a fixed voltage on the selected drain line. |

**Crossbar Array Architecture Based on Memtransistor Devices**

Crossbar array (CAA) architectures utilizing memtransistor devices can be configured in two ways for Multiplication and Accumulation (MAC) operations. In the structure shown in Fig. S1a, the gate terminal serves as a selector, with analogue input data applied to VD using the Ohmic region of the output curve. Alternatively, in Fig. S1b, VD acts as the selector, with analogue input applied to the gate terminal using the Ohmic region of the transfer curve. When constructing a vector-matrix multiplier (VMM) with three-terminal memtransistor devices, the integration density and structural complexity increase compared to two-terminal devices. However, this design mitigates crosstalk from sneak currents. By leveraging the Ohmic region, analogue data inputs enable MAC operations, unlike two-terminal devices where nonlinear characteristics necessitate time-domain approaches for MAC computation. For two-terminal memristor-based VMMs, resistance is determined during fabrication; low read currents enable low-power operation but complicate weight differentiation, whereas high read currents facilitate weight distinction at the cost of increased power consumption, presenting a trade-off. In contrast, ionic memtransistors allow read current control via the gate terminal without read disturbance (data degradation during reading), offering flexibility in managing power consumption and compatibility with CMOS operating environments.

| 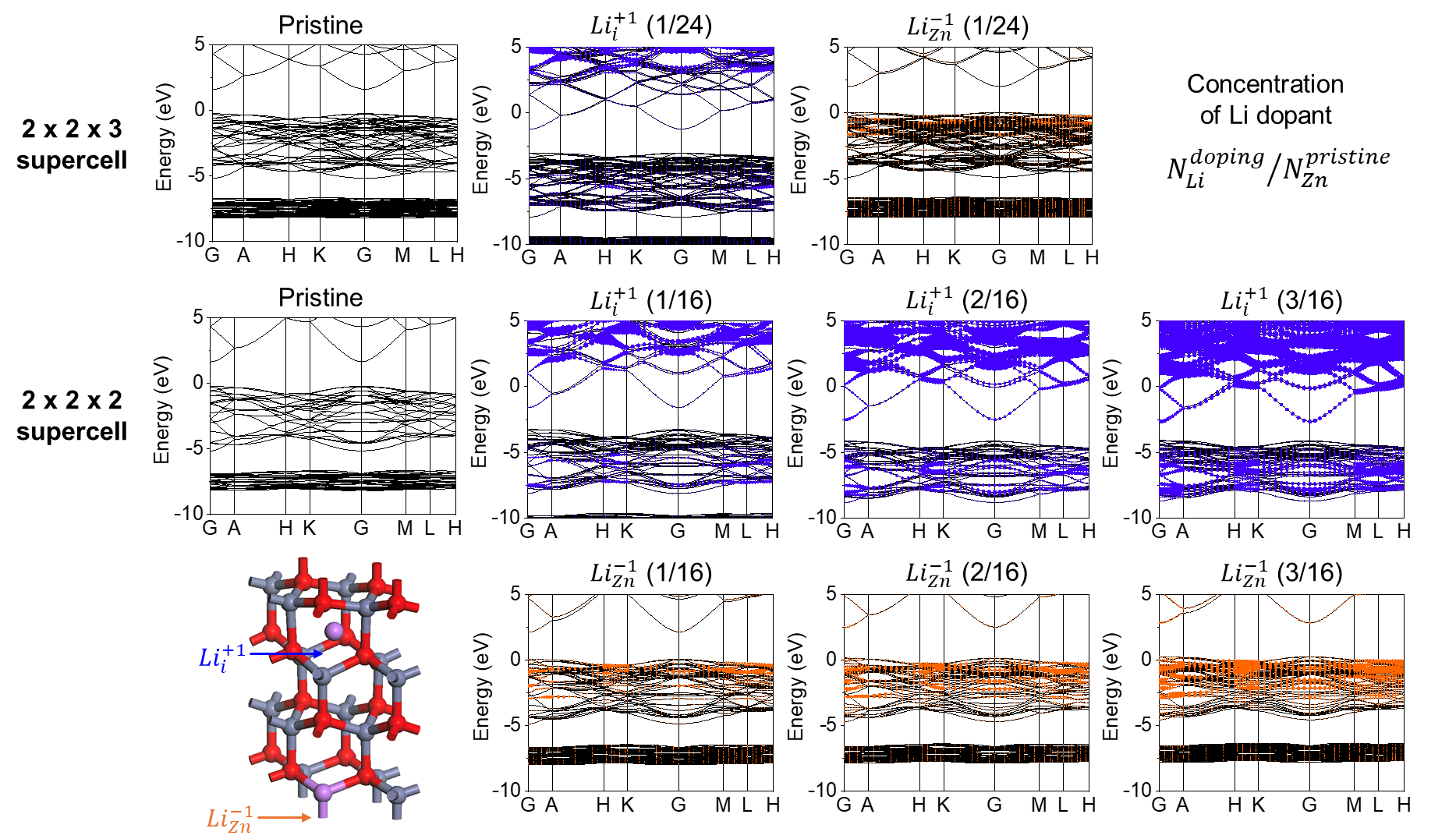 |
| --- |
| **Figure S2. Electronic structure analysis of Li-doped ZnO with varying dopant concentrations and configurations.** Band structures were calculated using 2×2×3 and 2×2×2 ZnO supercells to explore Li doping effects. Pristine ZnO (top left) exhibits a direct bandgap at the Γ point. Interstitial Li (${Li}_{i}^{+1}$) at 1/24 concentration (top center) lowers the conduction band minimum (CBM) below the Fermi level, signaling n-type doping. Substitutional Li (${Li}_{Zn}^{-1}$) at the same concentration (top right) raises the CBM. The middle row shows enhanced n-type behavior as ${Li}_{i}^{+1}$ concentration rises from 1/16 to 3/16, with denser states at the Fermi level. The bottom row reveals p-type evolution with increasing ${Li}_{Zn}^{-1}$ concentration, shifting the valence band maximum (VBM) upward. Blue and orange coloring denotes states with strong Li contributions, increasing in the conduction band for ${Li}_{i}^{+1}$ and valence band for ${Li}_{Zn}^{-1}$ with higher Li content. |

| 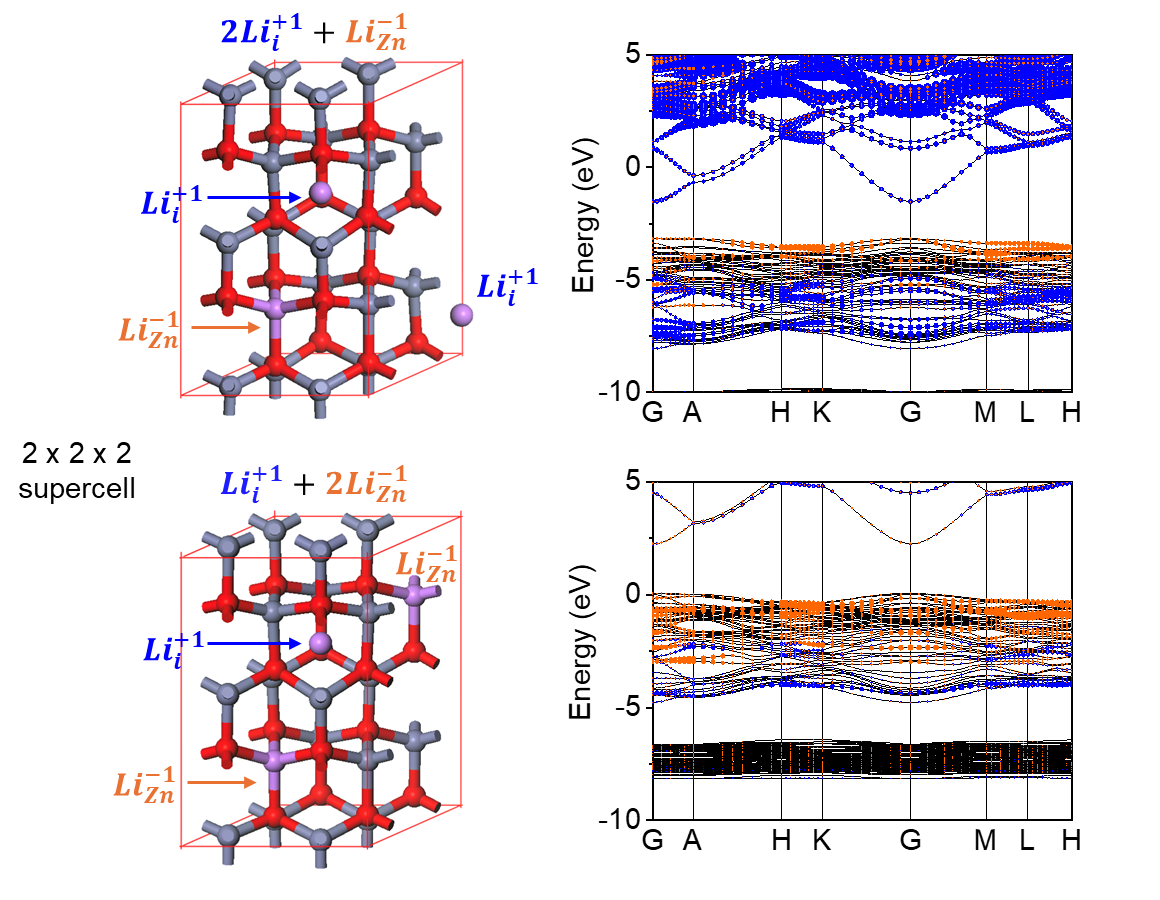 |
| --- |
| **Figure S3. Band structure analysis of competing Li defect configurations in ZnO.** Detailed investigation of electronic structure when both ${Li}_{i}^{+1}$ and ${Li}_{Zn}^{-1}$ defects coexist in different ratios within a 2×2×2 ZnO supercell. The top panel shows the case where ${Li}_{i}^{+1}$ defects dominate ($2{Li}_{i}^{+1}+{Li}_{Zn}^{-1}$), resulting in a net n-type character. The corresponding band structure (right) exhibits metallic behavior with multiple bands crossing the Fermi level. The bottom panel presents the opposite scenario (${Li}_{i}^{+1}+2{Li}_{Zn}^{-1}$) where p-type character prevails, leading to a distinctly different electronic structure. The partial density of states analysis reveals that the contribution of Li states increases in the conduction band for ${Li}_{i}^{+1}$ and in the valence band for ${Li}_{Zn}^{-1}$ (blue and orange coloring, respectively), maintaining similar characteristics as observed in single-type Li doping cases. These calculations demonstrate how the relative concentration of different Li defect types can tune the electronic properties of the material, directly impacting the device's switching characteristics. |

| 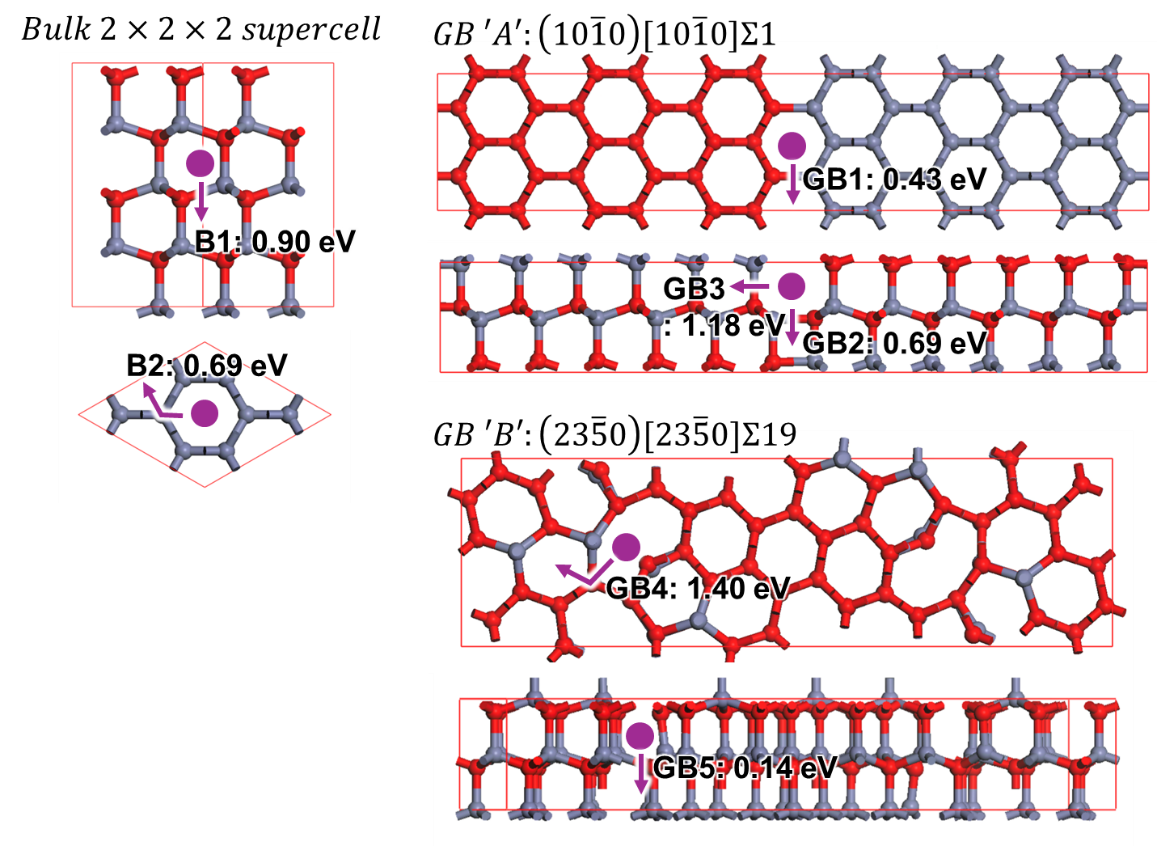 |
| --- |
| **Figure S4. Comprehensive analysis of Li-ion migration in both bulk ZnO and along grain boundaries.** The bulk 2×2×2 supercell calculations reveal two primary migration paths (B1 and B2) with relatively high barriers of 0.90 eV and 0.69 eV, respectively. Along grain boundaries, five distinct pathways (GB1-GB5) were identified, with barriers ranging from 0.14 eV to 1.40 eV. The *GB ‘A’* boundary shows a favorable GB1 path with a 0.43 eV barrier, while GB5 at the *GB ‘B’* boundary presents the lowest barrier (0.14 eV) among all pathways, attributed to its more open structure. The significantly lower migration barriers along certain grain boundaries compared to bulk diffusion pathways suggest that Li-ion transport occurs preferentially along GBs under the applied electric field. This selective transport mechanism through lower energy pathways explains how the device can achieve reliable switching operation at relatively low voltages despite the long channel length. |

| 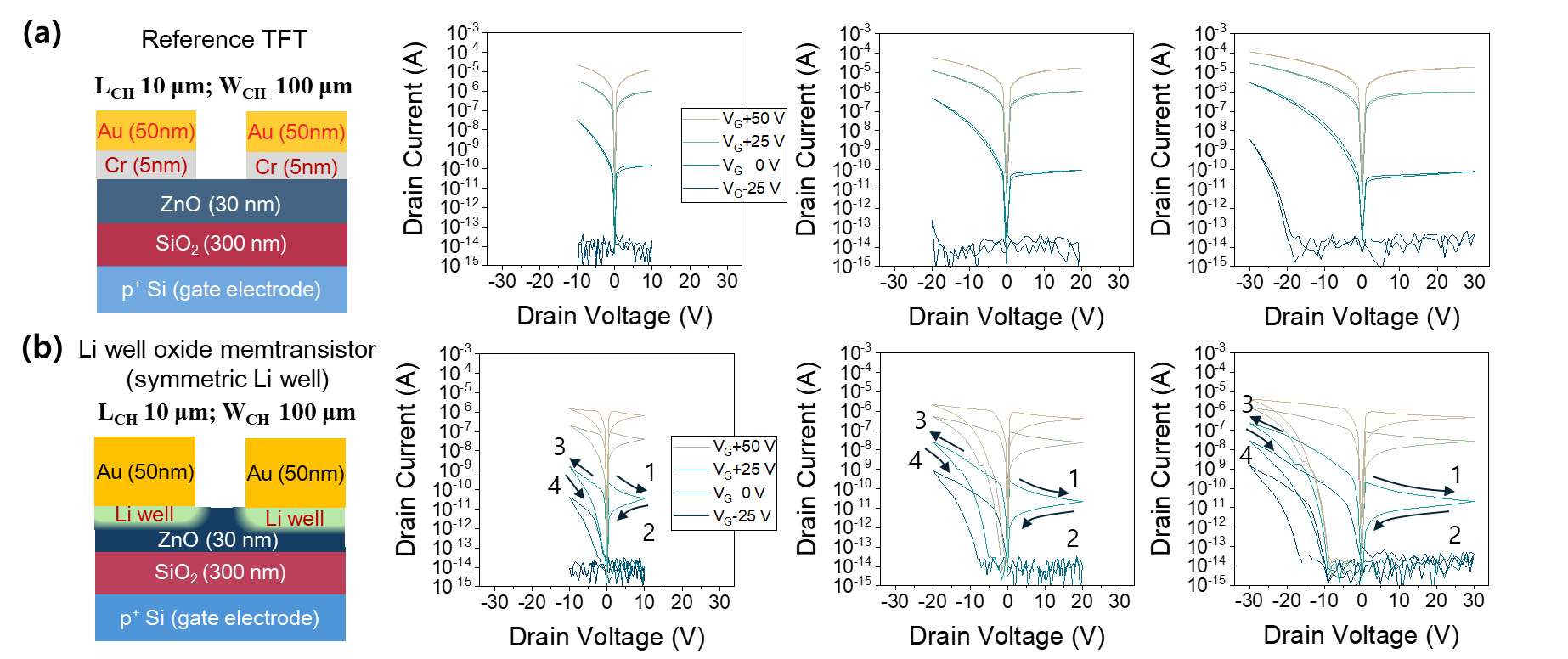 |
| --- |
| **Figure S5. Comparison of output curves with increasing V_DS_ for reference TFT and symmetric LWOM after 2-minute bake at 230 °C.** a) Output curves of the reference TFT with V_DS_ sweeps of 10 V, 20 V, and 30 V. b) Output curves of the symmetric LWOM with V_DS_ sweeps of 10 V, 20 V, and 30 V. Black arrows and numbers indicate the sweep sequence. |

| 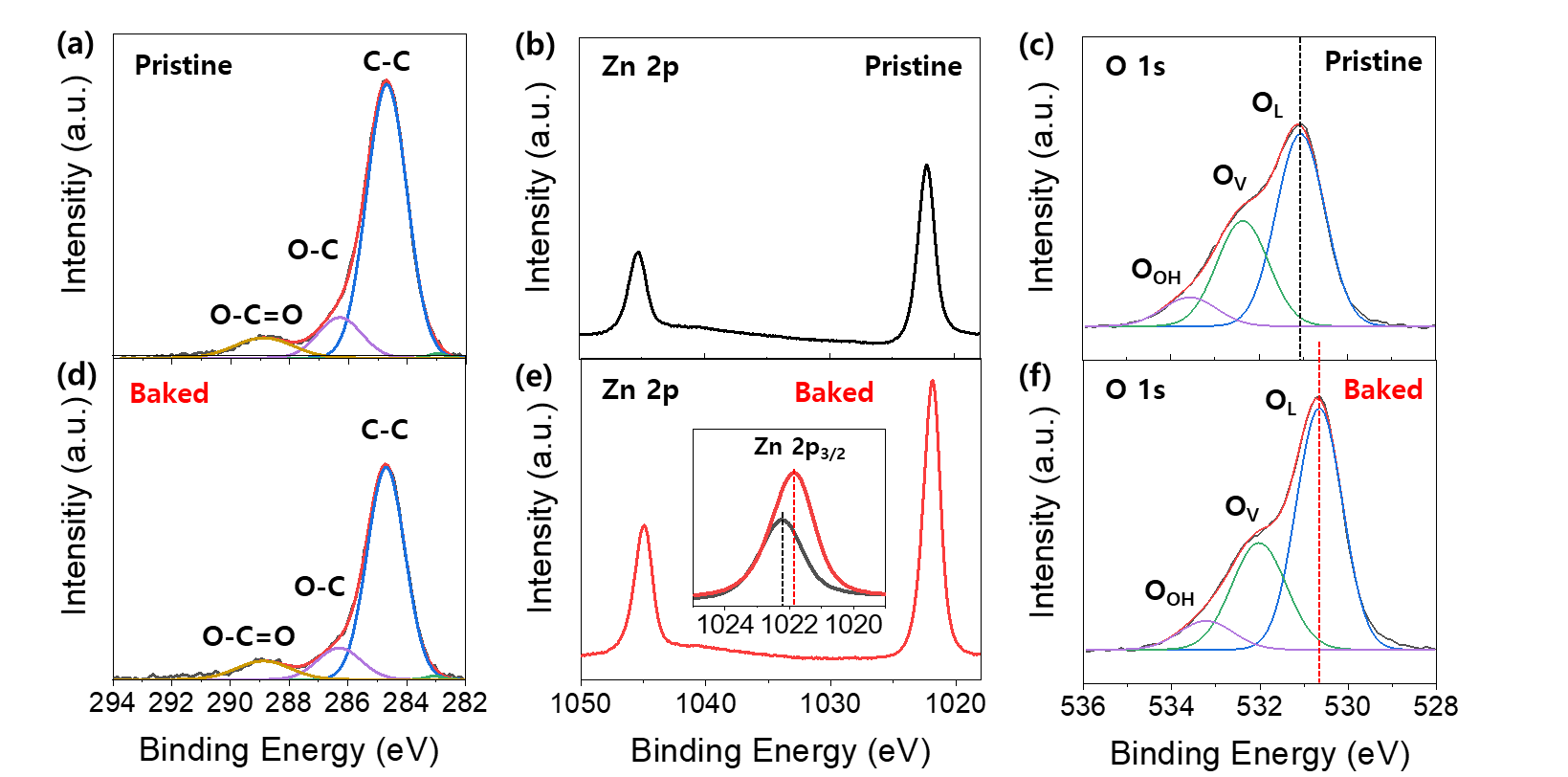 |
| --- |
| **Figure S6. Chemical changes in ZnO film before and after bake process**. a)-c), C 1s, Zn 2p, and O 1s XPS spectra from the pristine ZnO surface. d)-f), C 1s, Zn 2p, and O 1s XPS spectra from the ZnO surface after a 20-minute bake at 230 °C. Inset in the Zn 2p spectrum shows an enlarged, overlapped view of the Zn 2p_3/2_ peak for pristine (black) and baked (red) samples. Dotted lines in **c**, **e**, and **f** mark peak energy positions. Post-bake ZnO films exhibit reduced C 1s peak intensity, increased Zn 2p and lattice oxygen peak intensities compared to pre-bake, indicating thermal decomposition of residual ligands (or photoresist) and increased oxidation from atmospheric oxygen reaction. A 0.4 eV redshift in C 1s, Zn 2p, and O 1s peaks suggests a ~0.4 eV shift of the ZnO Fermi level toward the valence band maximum after heat treatment. |

| 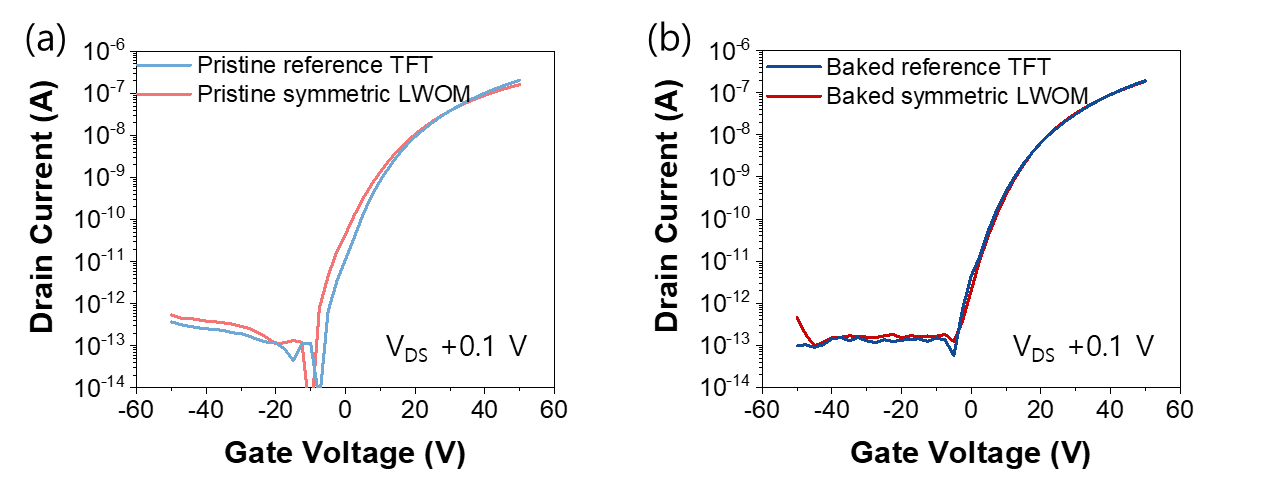 |
| --- |
| **Figure S7. Comparison of transfer curves before and after bake for reference TFT and symmetric LWOM.** a) Transfer curves of pristine reference TFT and symmetric LWOM devices. b) Transfer curves of reference TFT and symmetric LWOM devices after a 20-minute bake at 230 °C. Both devices exhibit identical turn-on voltages. |

| **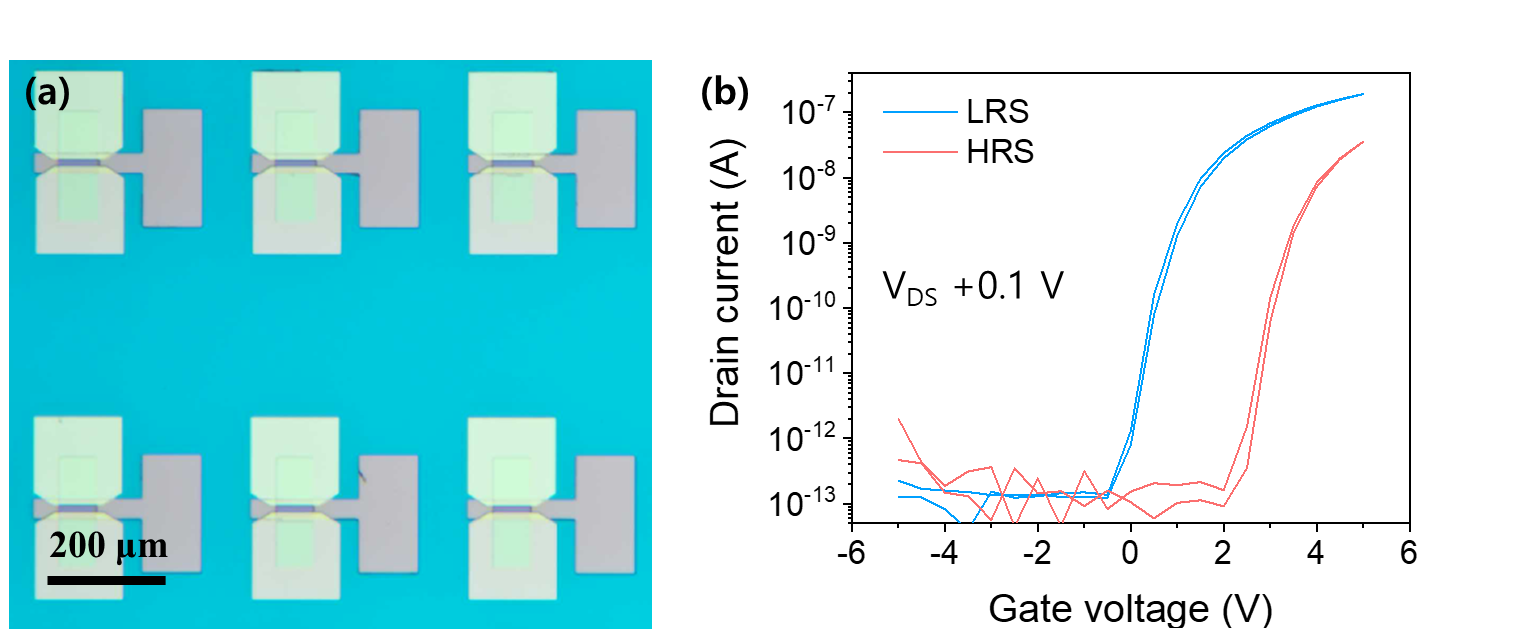** |
| --- |
| **Figure S8. Transfer curves of HRS and LRS for a local gate LWOM device switched with V_DS_=3 V (DC Sweep).** a) An optical microscope image of an asymmetric LWOM device with a local gate structure. b) Transfer curves for HRS and LRS states show no hysteresis loop during V_GS_ sweeps. |

| 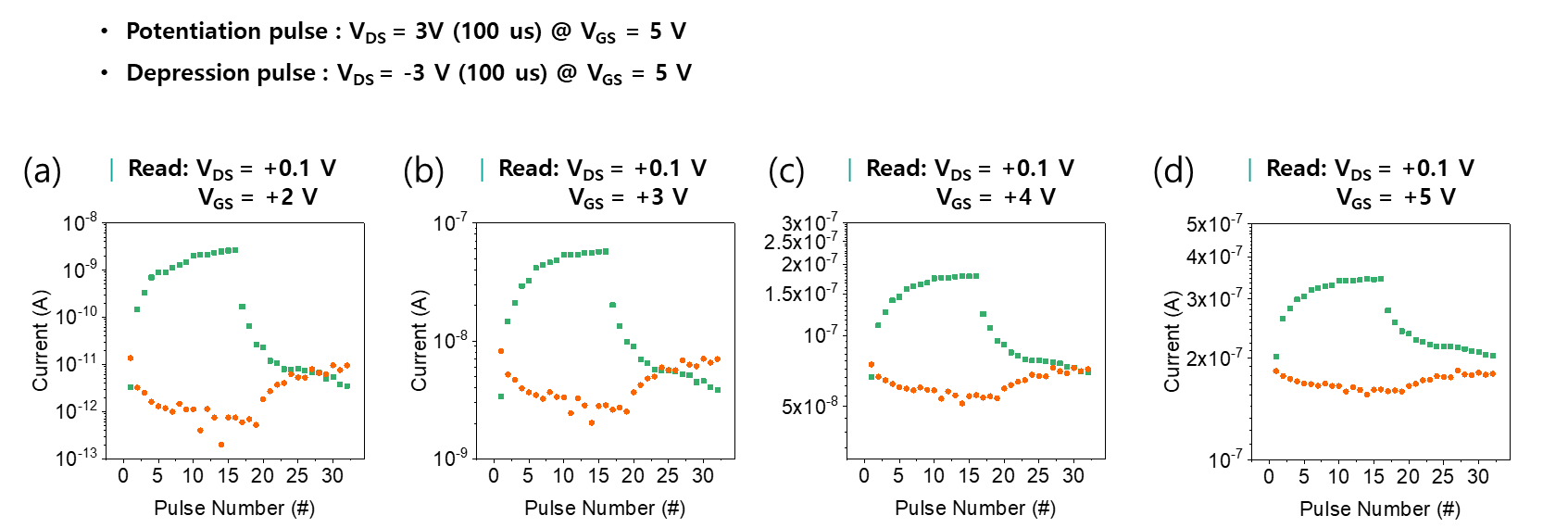 |
| --- |
| **Figure S9. Comparison of weight update curves with varying read V_GS_ voltages.** a)-d) 16-state LTP/LTD curves read at V_DS_=0.1 V with V_GS_ increasing from +2 V to +5 V. Green and orange curves represent weight updates with the Li well as source and drain, respectively, showing opposite weight update directions under identical conditions. As read V_GS_ increases, the current change ratio during weight updates decreases, while the absolute current difference increases. |

| 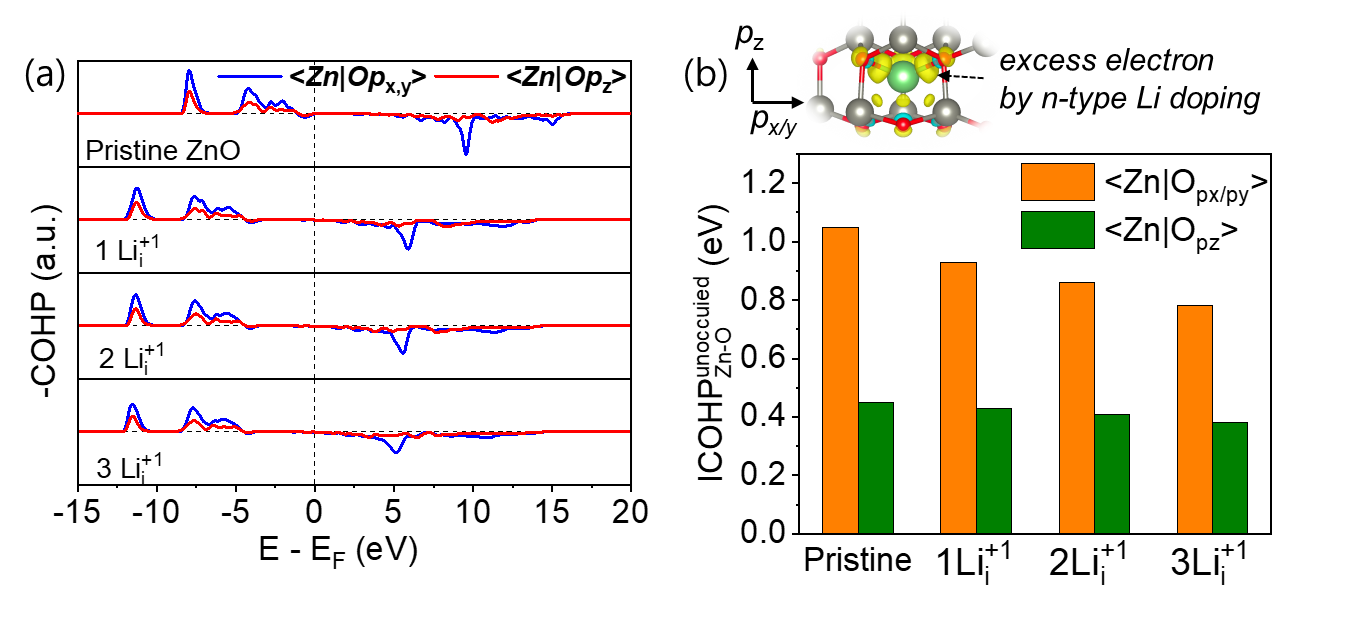 |
| --- |
| **Figure S10. Analysis of orbital interactions between Zn and O_2p_ states upon Li doping via Crystal Orbital Hamiltonian Population (COHP).** a) Evolution of orbital overlap between Zn and O_2p_ states with increasing ${Li}_{i}^{+1}$ concentration, showing COHP plots for pristine ZnO and systems with 1-3 ${Li}_{i}^{+1}$ dopants. The analysis separates the contributions into Zn-O_px/py_ (<Zn\|O_px/py_>) and Zn-O_pz_ (<Zn\|O_pz_>) interactions, revealing a systematic reduction in unoccupied states density, particularly in the O_px/py_ directions with increasing Li doping. b) Quantitative analysis through integrated COHP values of unoccupied states for O_px/py_ and O_pz_ orbitals. The results confirm that excess electrons introduced by n-type Li interstitial doping preferentially fill the unoccupied O_px/py_-derived states in the a-b plane, directly explaining the systematic reduction in peak B intensity observed in O K-edge EELS spectra. |


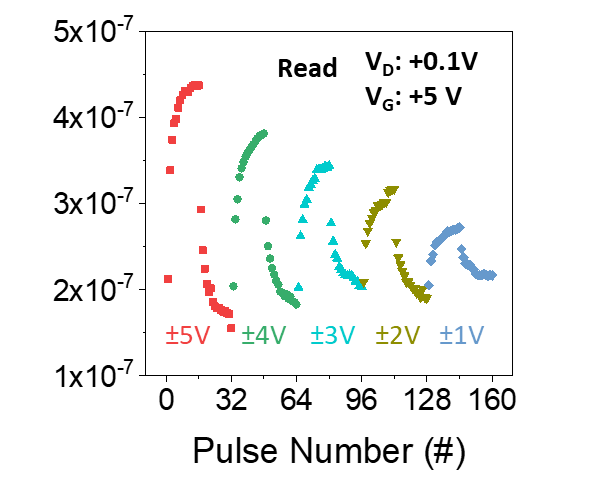


**Figure S11. Weight update characteristics.** 16-state LTP/LTD curves obtained by weight updates with V_GS_ = +5 V and V_DS_ = 1 V to 5 V (100 μs), followed by reads at V_DS_ = +0.1 V with V_GS_ = +5 V, respectively.

| \| 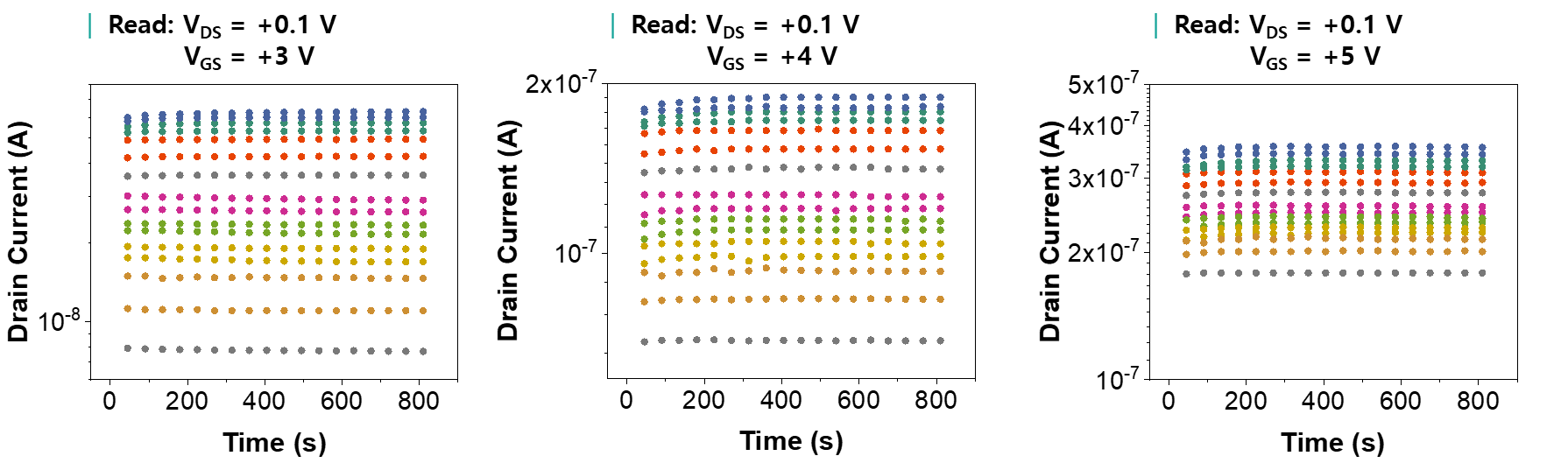 \| \| --- \| \| **Figure S12. Retention Characteristics of Each of the 16 States.** Retention data collected at four read V_GS_ values (+2 V, +3 V, +4 V, +5 V), with results for +3 V, +4 V, and +5 V shown. \|   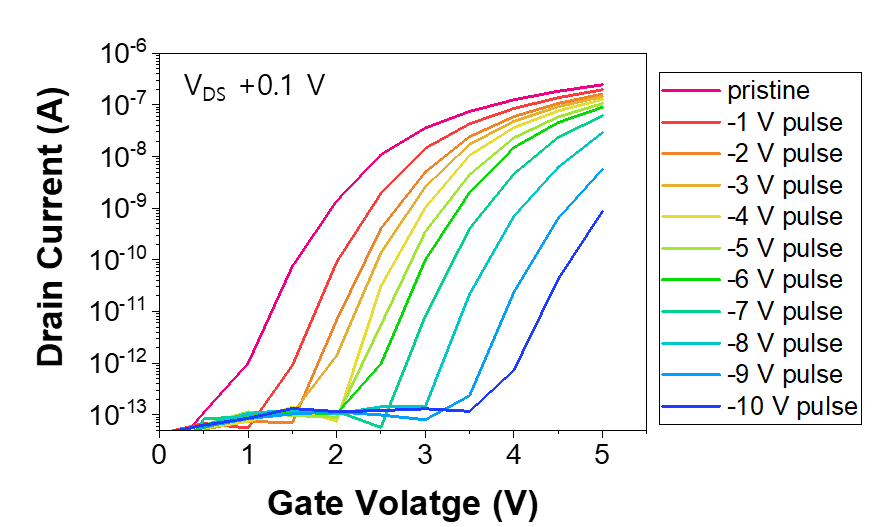 |
| --- | --- | --- |
| **Figure S13. Transfer curve changes with write voltage magnitude.** The turn-on voltage shift varies with write voltage magnitude, increasing the memory window as the write voltage increases. |

**Figure S14. Pulse-mode endurance with read after every switching event.** Endurance characteristics of an LWOM device measured up to 1.1 × 10⁴ cycles in pulse mode, with a read operation performed after each switching event. Write pulses were applied to the drain (±5 V) and gate (+5 V) with a pulse width of 1 ms, while read operations were conducted at V_DS_ ​ = +0.1 V and V_GS_ ​ = +3 V. No significant degradation in the ON/OFF ratio or current levels was observed throughout the test, and the gradual current decrease seen in Figure 4i at higher cycle counts was not present in this separate device.


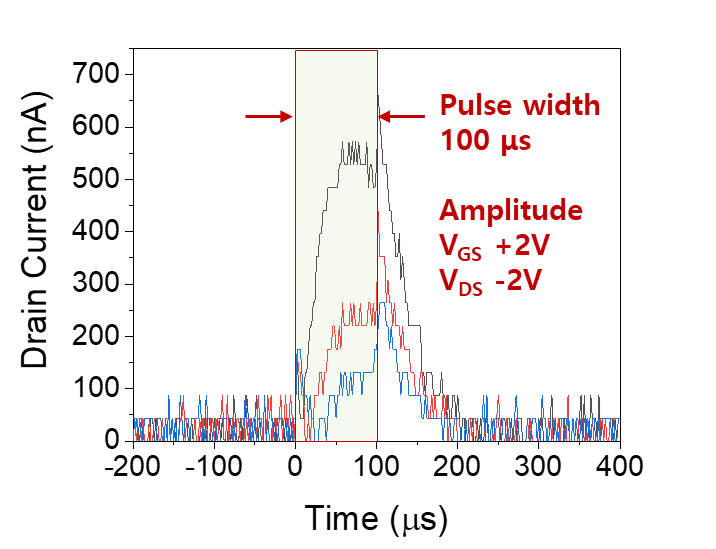


**Figure S15. Weight update pulse measurement.** Measured drain current waveforms during weight update pulses (width: 100 μs, V_GS_ ​ = +2 V, V_DS_ ​ = –2 V) used to calculate the power consumption for each conductance transition presented in Figure 4j. The current was indirectly measured by connecting a 1 MΩ external resistor in series with the LWOM and monitoring the voltage drop across it using an oscilloscope with a sampling interval of 2 μs. A transient current peak was observed immediately after the end of the programming pulse, which is attributed to capacitive discharge from the device and wiring, as well as transient responses of the measurement circuitry, rather than actual device conduction. Accordingly, for power calculation, only the current within the time window of the applied programming pulse was integrated, excluding post-pulse artifacts.


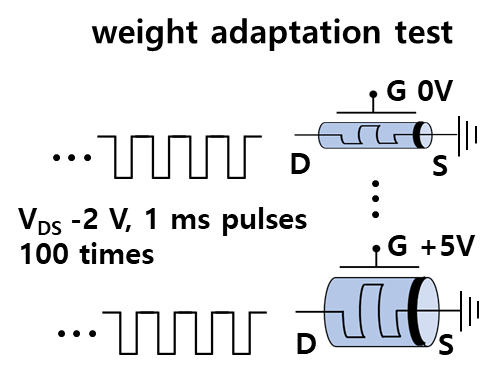


**Figure S16. Schematic of the weight adaptation test.** Illustration of the measurement protocol used to control and examine the saturation point of weight updates in the LWOM device. The gate voltage V_GS_ ​was stepped from 0 V to 5 V in 1 V increments, and for each V_GS_ ​ step, 100 drain pulses (V_DS_ ​ = –2 V, pulse width = 1 ms) were applied. This process allowed Li-ion redistribution at the interface until a conductance saturation point was reached, after which V_GS_ ​ was increased to the next step and the procedure repeated. The resulting V_GS_-controlled saturation behavior is presented in Figure 4k.


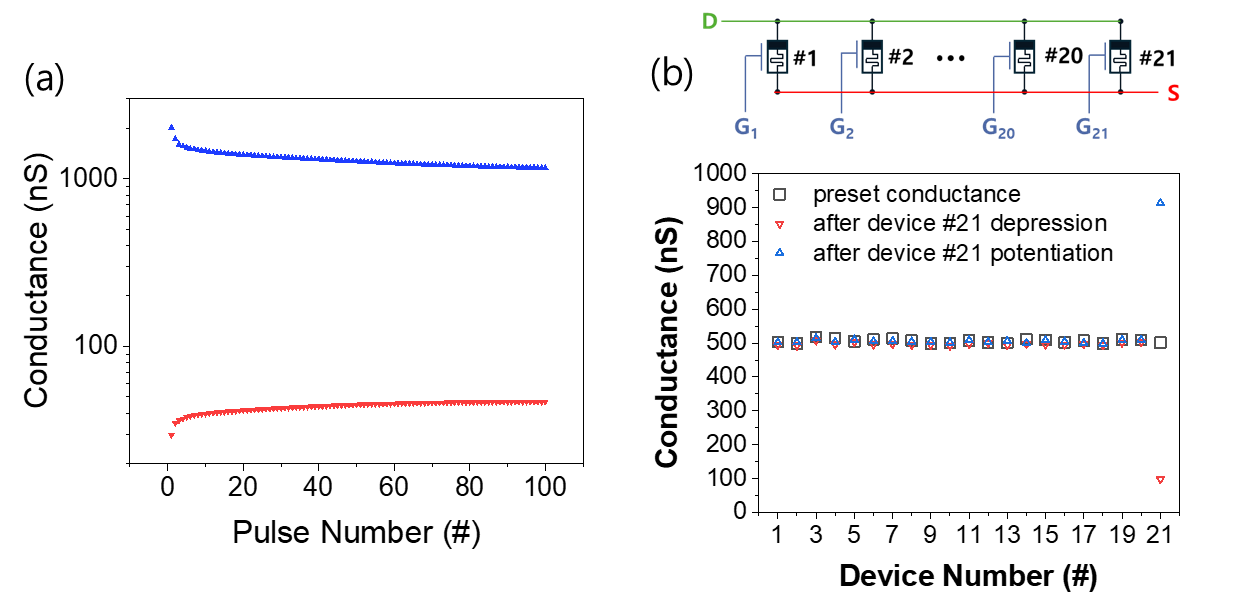


**Figure S17. Saturation-based selective programming in a crossbar array.** (a) Saturation curves for LTP (from full HRS) and LTD (from full LRS) obtained by applying 100 pulses (width: 100 μs, V_GS_ ​ = 0 V, V_DS_ ​ = ±2 V). (b) Disturbance test in a 21-device parallel configuration. The black plot shows the initial set conductance values of devices #1–#21. The red plot shows the conductance values after applying 17 LTD update pulses (width: 100 μs, V_GS_ ​ = +2 V, V_DS_​ = –2 V) to device #21, and the blue plot shows the conductance values after applying 72 LTP update pulses (width: 100 μs, V_GS_ ​ = +2 V, V_DS_ = +2 V) to the same device.

| 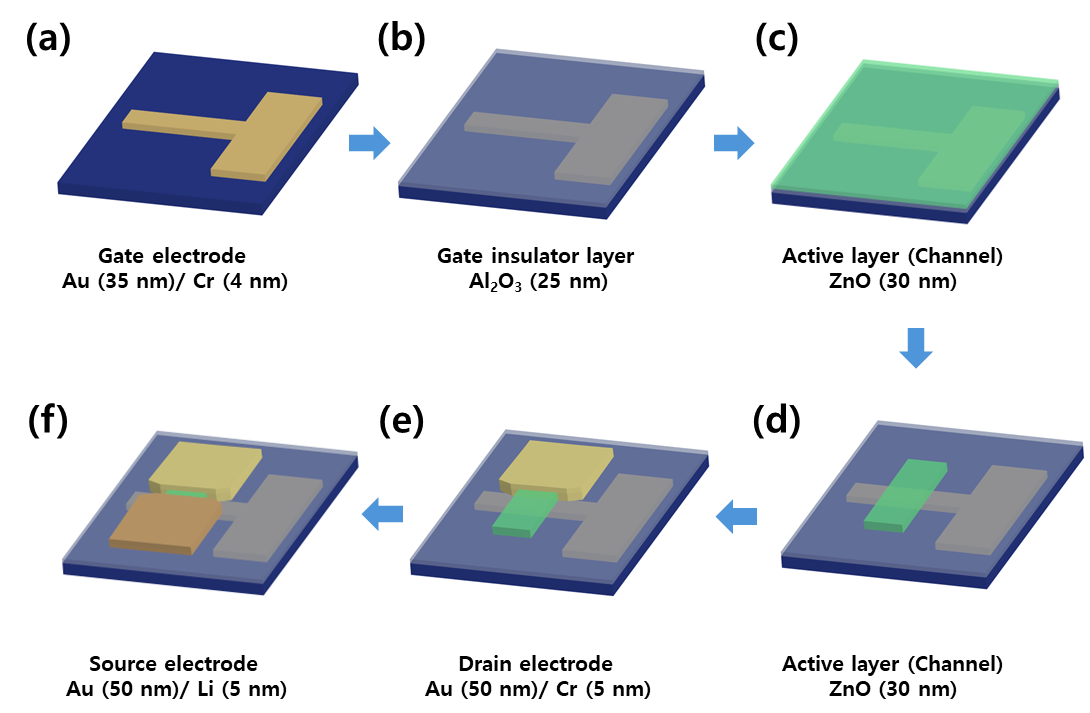 |
| --- |
| **Figure S18. Fabrication schematic of a single asymmetric LWOM device.** a) Formation of a local gate electrode. b)-c) Deposition of a 25 nm Al₂O₃ layer and a 30 nm ZnO layer via ALD. d) Channel definition by etching. e) Drain electrode fabrication with a Cr adhesion layer. f) Source electrode formation with a Li adhesion layer, completing the device. |

| 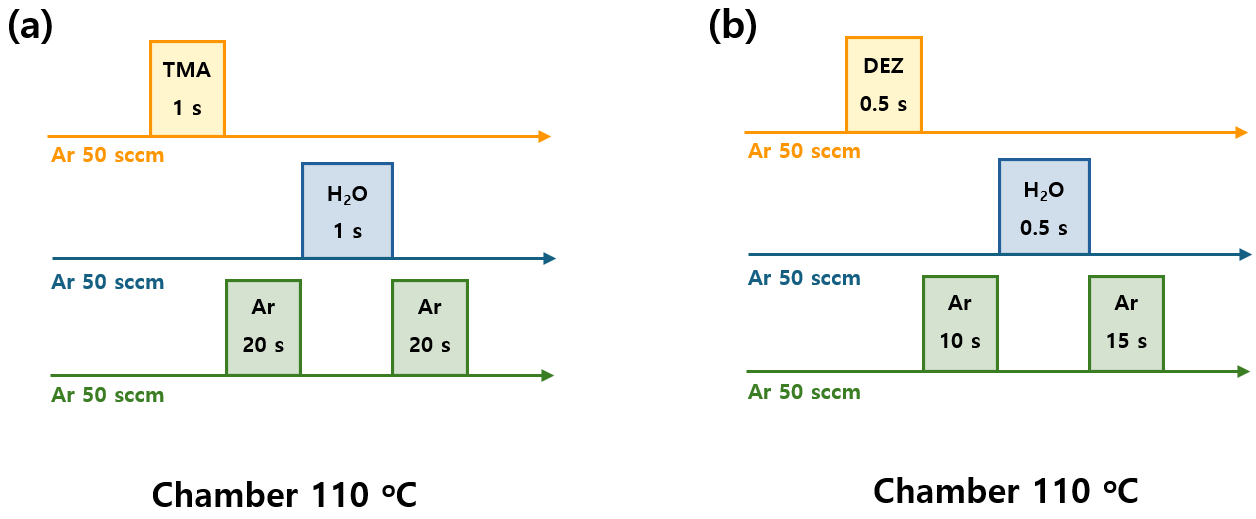 |
| --- |
| **Figure S19. ALD Cycle Sequences for Al₂O₃ and ZnO.** a) Al₂O₃ deposition (200 cycles, 25 nm) using TMA (1 s pulse) → Ar (20 s purge) → H₂O (1 s pulse) → Ar (20 s purge), with a GPC of 1.25 Å. b) ZnO deposition (200 cycles, 30 nm) using DEZ (0.5 s pulse) → Ar (10 s purge) → H₂O (0.5 s pulse) → Ar (15 s purge), with a GPC of 1.5 Å. |

| 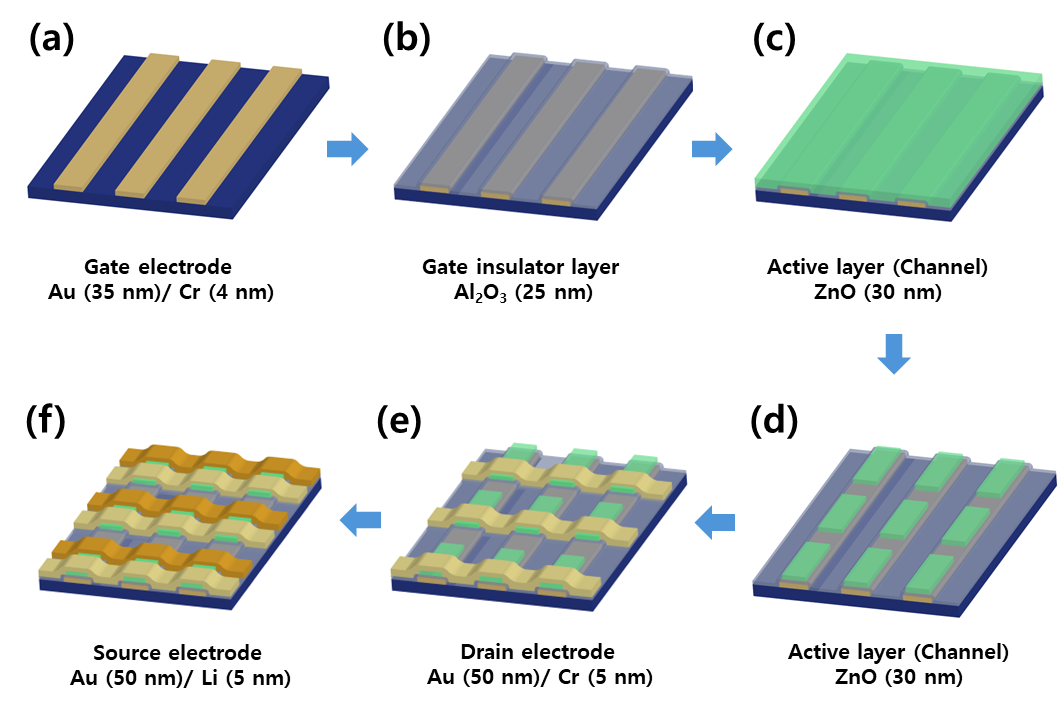 |
| --- |
| **Figure S20. Fabrication schematic of a 21 × 21 crossbar array device.** a) Fabrication of 21 gate electrodes. b)-c) Deposition of 25 nm Al₂O₃ and 30 nm ZnO via ALD. d) Definition of 441 channels by etching. e) Formation of 21 drain electrodes with a Cr adhesion layer. f) Fabrication of 21 source electrodes with a Li adhesion layer, completing the 21 × 21 crossbar array. |

| 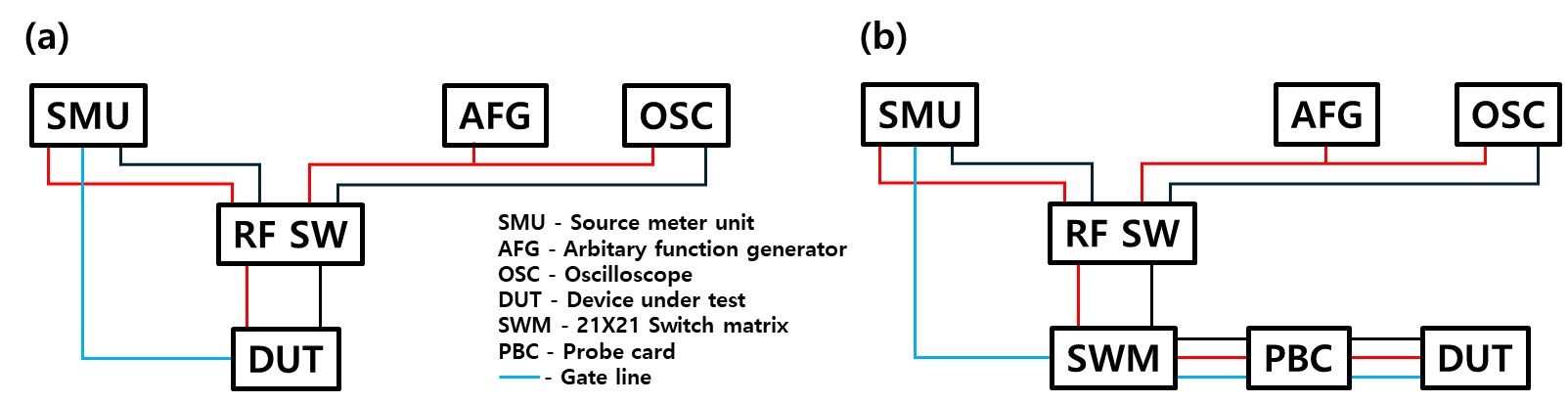 |
| --- |
| **Figure S21. Circuit diagram of the device measurement system.** a) Signal path from the source meter unit (SMU) through the arbitrary function generator (AFG) and oscilloscope (OSC) to the RF switch (RF SW), then to the device under test (DUT). Gate vias connect directly from the SMU to the DUT. b) For 21 × 21 crossbar array measurements, the RF SW connects to a switch matrix (SWM), linked to a probe card (PBC) with 63 tips physically contacting the DUT. |

| 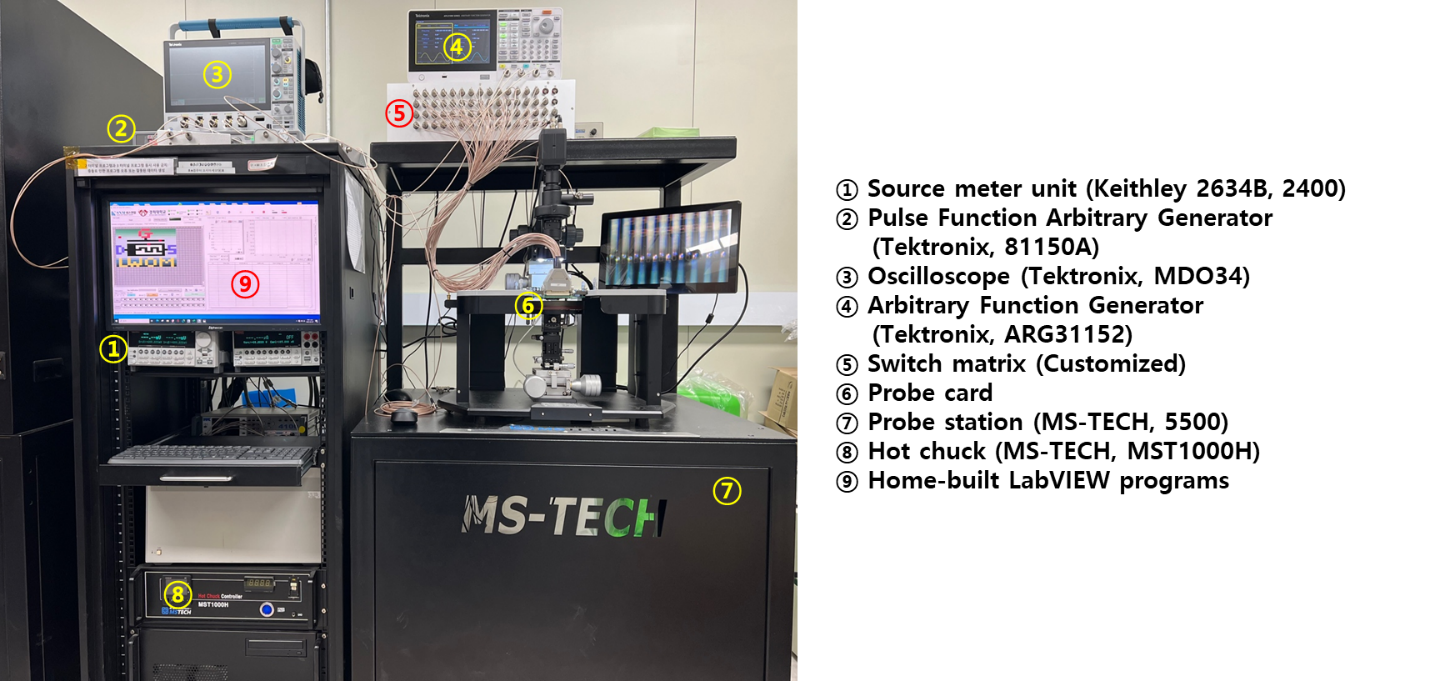 |
| --- |
| **Figure S22. Photograph of the 21 × 21 crossbar array device measurement system.** |

| 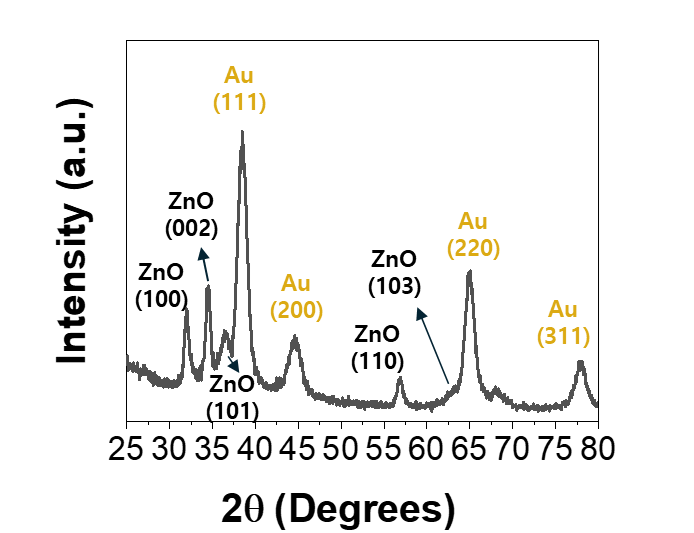 |
| --- |
| **Figure S23. XRD spectrum of a baked Au (10 nm)/Li (6 nm)/ZnO (30 nm) thin film.** |

Table S1. Comparison of key performance metrics for three types of memtransistor devices: ion-migration, ferroelectric, and charge-trap (floating gate).

| Ref | This work | 1 | 2 | 3 | 4 | 5 | 6 | 7 | 8 | 9 | 10 | 11 | 12 | 13 | 14 | 15 | 16 |
| --- | --- | --- | --- | --- | --- | --- | --- | --- | --- | --- | --- | --- | --- | --- | --- | --- | --- |
| Crossbar array | 21 x 21 | - |  | - | - | - | - | - | - | - | 8 x 8 | 9 x 7 | 4 x 4 | - | - | - | 9 x 2 |
| Writing  terminal | V_D_, V_G_ | V_D_, V_G_ | V_D_, V_G_ | V_D_, V_G_ | V_D_, V_G_ | V_D_, V_G_ | V_G_ | V_G_ | V_G_ | V_G_ | V_G_ | V_G_ | V_D_, V_G_ | V_D_ | V_D_ | V_G_ | V_G_ |
| Multi-bit | Yes | Yes | Yes | Yes | Yes | Yes | Yes | Yes | Yes | Yes | Yes | Yes | Yes | Yes | Yes | Yes | Yes |
| Transistor's on/off ratio | 10^6^ | 10^6^ | 10^3^ | - | - | - | - | 10^6^ | 10^5^ | 10^6^ | 10^4^ | 10^3^ | 10^7^ | 10^3^ | 10^7^ | 10^9^ | 10^6^ |
| On/off ratio | 10^5^ ~ 10^6^ | 10^4^ | 10^3^ | 10^3^ | 10^4^ | 10^3^ | - | 10^6^ | 10^5^ | 10^6^ | 10^6^ | 10^3^ | 10^8^ | 10^7^ | 10^6^ | 10^4^ | 10^8^ |
| Weight update speed | 100 μs | 20 ~  50 ms | 1 ms | 10 ms | 50 ~  100 ms | 50 ms | 40 ns | 100 μs | 1 μs | 500 ms | 500 μs | 10 ns | 40 ns | 20 ~  1000 ms | 100 ms | 100 μs | 5 μs |
| Writing voltage | 3 V | 20 V | 30 V | 3 V | 10 V | 30 ~  40 V | 3 V | 1 ~  4 V | 2 ~  4 V | 6 V | 4.5 V | 4.5 V | 10 ~  13 V | 8 ~  10 V | 6 V | 15 ~  18 V | 3 V |
| Retention | > 8 x 10^4^ s | > 4 x 10^4^ s | > 8 x 10^4^ s | 100 s | 1000 s | 600 s | 1.5 x 10^3^ s | > 4 s | - | 1000 s | 5 x 10^4^ s | 10^5^ s | > 10^4^ s | 1000 s | 10^5^ s | 3 x 10^4^ s | 10^4^ s |
| Endurance | >1.2 x 10^6^ (pulse) | 100 (DC) | 250 (DC) | 100 (DC) | 5000 (pulse) | 100 (DC) | 10^8^ (pulse) | - | 1.4 x 10^4^ (pulse) | 2 x 10^5^ (pulse) | 10^5^ (pulse) | 10^4^ (pulse) | 10^5^ (pulse) | 10^3^ (pulse) | 10^4^ (pulse) | 10^4^ (pulse) | 10^4^ (pulse) |
| Tunnel oxide | - | - | - | - | - | - | - | - | - | - | - | - | 2D h-BN | 2D h-BN | Al_2_O_3_ | Al_2_O_3_ | Al_2_O_3_ |
| Floating gate | - | - | - | - | - | - | - | - | - | - | - | - | Graphene | Graphene | Graphene | ZnO | Pt |
| Gate oxide | Al_2_O_3_ | SiO_2_ | Al_2_O_3_ | SiO_2_ | Al_2_O_3_/  TiO_2_ | SiO_2_ | HZO (Ferro) | HfO_2_ (Ferro) | HZO (Ferro) | HZO (Ferro) | Si:HfO_2_ (Ferro) | HfO_2_ (Ferro) | - | - | - | Al_2_O_3_ | - |
| Channel | ZnO | IGZO | MoS_2_ | HxK₁₋ₓGaSb₂ | IGZO | WSe_2_ | WOx | a-In_2_OSe_3_ | 2D SnS_2_ | IGZO | p-Si | p-Si | MoS_2_ | MoO_3_/  MoS_2_ | ZnO | ZnO | MoS_2_ |
| Mechanism | Ion migration | | | | | | Ferroelectric  (FeFET) | | | | | | Charge trap  (Floating gate) | | | | |

Table S2. d-spacing of the ZnO film obtained from the XRD spectrum (Figure S18) and the crystallite size calculated using the Scherrer equation.

|  | 100 | 002 | 110 |
| --- | --- | --- | --- |
| Peak (2θ) | 32 | 34.51 | 56.85 |
| d (nm) | 0.58 | 0.601 | 0.8 |
| Grain size (nm) | 14.89 | | |

**References**

[1] S. Nam, D. Kang, S.-P. Jeon, et al., “Contact‑Engineered Oxide Memtransistors for Homeostasis‑Based High‑Linearity and Precision Neuromorphic Computing,” *Small* 21, no. 7 (2025): 2409510. <https://doi.org/10.1002/smll.202409510>

[2] H.-S. Lee, V. K. Sangwan, W. A. G. Rojas, et al., “Dual-Gated MoS_2_ Memtransistor Crossbar Array,” *Advanced Functional Materials* (2020): 2003683, [*https://doi.org/10.1002/adfm.202003683*](https://doi.org/10.1002/adfm.202003683)

[3] J. Bae, M. Kang, J. Lee, et al., “Cation‑Eutaxy‑Enabled III–V‑Derived van der Waals Crystals as Memristive Semiconductors,” *Nature Materials* 23, no. 10 (2024): 1402–1410. <https://www.nature.com/articles/s41563-024-01986-x>

[4] H. Park, S. Oh, S.-H. Jeong, et al., “Dual‑Terminal Stimulated Heterosynaptic Plasticity of IGZO Memtransistor with Al₂O₃/TiO₂ Double‑Oxide Structure,” *ACS Applied Electronic Materials.* 4, no. 6 (2022): 2923–2932. <https://pubs.acs.org/doi/full/10.1021/acsaelm.2c00393>

[5] G. Ding, B. Yang, R.-S. Chen, et al., “Reconfigurable 2D WSe₂‑Based Memtransistor for Mimicking Homosynaptic and Heterosynaptic Plasticity,” *Small* 17, no. 41 (2021): 2103175. [https://onlinelibrary.wiley.com/doi/full/10.1002/smll.202103175](https://onlinelibrary.wiley.com/doi/full/10.1002/smll.202103175%20)

[6] M. Halter, L. Bégon‑Lours, V. Bragaglia, et al., “Back‑End, CMOS‑Compatible Ferroelectric Field‑Effect Transistor for Synaptic Weights,” *ACS Applied Materials & Interfaces* 12, no. 15 (2020): 17725–17732. https://doi.org/10.1021/acsami.0c00877

[7] K. Liu, T. Zhang, B. Dang, et al., “An optoelectronic synapse based on α‑In₂Se₃ with controllable temporal dynamics for multimode and multiscale reservoir computing,” *Nature Electronics* 5, no. 10 (2022): 761–773. https://doi.org/10.1038/s41928-022-00847-2

[8] C.-M. Song, D. Kim, S. Lee and H.-J. Kwon, “Ferroelectric 2D SnS₂ Analog Synaptic FET,” *Advanced Science* 11, no. 16 (2024): e2308588. https://doi.org/10.1002/advs.202308588

[9] F. Xi, Y. Han, M. Liu, et al., “Improvement of polarization switching in ferroelectric transistor by interface trap reduction for brain-inspired artificial synapses,” *ACS Applied Materials & Interfaces* 13, no. 27 (2021): 32385–32393. <https://doi.org/10.1021/acsami.1c07505>

[10] S. De, F. Müller, N. Laleni, et al., “Demonstration of Multiply‑Accumulate Operation With 28 nm FeFET Crossbar Array,” *IEEE Electron Device Letters* 43, no. 12 (2022): 2081–2084. https://doi.org/10.1109/LED.2022.3220947

[11] T. Soliman, S. Chatterjee, N. Laleni, et al., “First demonstration of in‑memory computing crossbar using multi‑level Cell FeFET,” *Nature Communications* 14, no. 1 (2023): 6348. <https://doi.org/10.1038/s41467-023-42110-y>

[12] Y. Tang, X. Yan, X. Liu, et al., “A Reliable All‑2D Materials Artificial Synapse for High Energy‑Efficient Neuromorphic Computing,” *Advanced Functional Materials* 31, no. 33 (2021): 2102302. <https://doi.org/10.1002/adfm.202011083>

[13] H. Zhu, Z. Wang, W. Ma, et al., “Surface Charge Transfer Doping Enabled Large Hysteresis in van der Waals Heterostructures for Artificial Synapse,” *ACS Materials Letters* 2, no. 6 (2020): 569–575. <https://doi.org/10.1021/acsmaterialslett.0c00531>

[14] Y. Wang, Z. Li, X. Luo, et al., “Wafer‑scale floating gate memristor array using 2D‑graphene/3D‑Al₂O₃/ZnO heterostructures for neuromorphic system,” *Applied Surface Science* 636 (2025): 162460. <https://doi.org/10.1016/j.apsusc.2025.162460>

[15] H. T. Nguyen, T. Y. Hwang and D. H. Kim, “Transparent Floating Gate Memory Based on ZnO Thin Film Transistor With Controllable Memory Window,” *IEEE Journal of the Electron Devices Society* 10 (2022): 281–285. <https://doi.org/10.1109/JEDS.2022.3159787>

[16] H. Li, Y. Liu, X. Zhang, et al., “8-bit states in 2D floating-gate memories using gate-injection mode for large-scale convolutional neural networks,” *Nature Communications* 15, no. 1 (2024): 4172. <https://doi.org/10.1038/s41467-025-58005-z>
